# Supplementary material for: Association of psychological distress, quality of life and costs with carpal tunnel syndrome severity: a cross-sectional analysis of the PALMS cohort
Source: BMJ Open. 2017 Nov 3;7(11):e017732. doi: 10.1136/bmjopen-2017-017732 (PMC5722090; doi:10.1136/bmjopen-2017-017732)
Supplement: Supplementary file 1 [file bmjopen-2017-017732supp001.pdf]

Supplementary File I:

Criteria for grading of nerve conduction studies:

Grade 0	Normal	No neurophysiological abnormality
Grade 1	Very mild	Median SAP digit II or III to wrist: < 50m/s and ≥40 m/s, Ulnar SAP>50m/s<br>Median DML APB to wrist: >4.0ms and Ulnar DML <=3.8ms<br>or<br>2 sensitive tests positive (ring finger 'double peak' SAP or palm/wrist – median/ulnar comparison or Lumbrical to interosseous latency increase)
Grade 2	Mild	Median SAP digit II or III to wrist SAP: < 40 m/sec<br>Median DML APB to wrist: < 4.5msec
Grade 3	Moderate	Median SAP digit II or III to wrist: present<br>Median DML APB to wrist: > 4.5msec and < 6.5 ms
Grade 4	Severe	Median SAP digit II or III to wrist: absent<br>Median DML APB to wrist: > 4.5 and < 6.5 ms
Grade 5	Very severe	Median DML APB to wrist : > 6.5 msec
Grade 6	Extremely severe	Median DML APB to wrist: unrecordable (< 0.2 mV peak to peak)

SAP: sensory action potential, DML: distal motor latency; APB: Abductor Pollicis Brevis; ms: milliseconds
