# Supplementary material for: Association of psychological distress, quality of life and costs with carpal tunnel syndrome severity: a cross-sectional analysis of the PALMS cohort
Source: BMJ Open. 2017 Nov 3;7(11):e017732. doi: 10.1136/bmjopen-2017-017732 (PMC5722090; doi:10.1136/bmjopen-2017-017732)
Supplement: Author's manuscript [file bmjopen-2017-017732.draft_revisions.pdf]

BMJ Open is committed to open peer review. As part of this commitment we make the peer review history of every article we publish publicly available.

When an article is published we post the peer reviewers' comments and the authors' responses online. We also post the versions of the paper that were used during peer review. These are the versions that the peer review comments apply to.

The versions of the paper that follow are the versions that were submitted during the peer review process. They are not the versions of record or the final published versions. They should not be cited or distributed as the published version of this manuscript.

BMJ Open is an open access journal and the full, final, typeset and author-corrected version of record of the manuscript is available on our site with no access controls, subscription charges or pay-per-view fees (http://bmjopen.bmj.com).

If you have any questions on BMJ Open's open peer review process please email editorial.bmjopen@bmj.com

BMJ Open

Association of psychological distress, quality of life and costs with carpal tunnel syndrome severity

Journal:	*BMJ Open*
Manuscript ID	bmjopen-2017-017732
Article Type:	Research
Date Submitted by the Author:	15-May-2017
Complete List of Authors:	Jerosch-Herold, Christina; University of East Anglia, Health Sciences<br>Houghton, Julie; University of East Anglia Faculty of Medicine and Health Sciences, Health Sciences<br>Blake, Julian; Norfolk and Norwich University Hospitals NHS Foundation Trust, Neurophysiology<br>Shaikh, Anum; University of Cambridge Department of Public Health and Primary Care<br>Wilson, Edward; University of Cambridge, Cambridge Centre for Health Services Research<br>Shepstone, Lee; University of East Anglia Faculty of Medicine and Health Sciences
**Primary Subject Heading**:	Health services research
Secondary Subject Heading:	Health economics, Mental health, General practice / Family practice, Neurology
Keywords:	Neurological pain < NEUROLOGY, Neurophysiology < NEUROLOGY, Hand & wrist < ORTHOPAEDIC & TRAUMA SURGERY, Musculoskeletal disorders < ORTHOPAEDIC & TRAUMA SURGERY, PRIMARY CARE, Depression & mood disorders < PSYCHIATRY

SCHOLARONE™
Manuscripts

For BMJ Open

Title

Association of psychological distress, quality of life and costs with carpal tunnel syndrome severity

Authors

Christina Jerosch-Herold[1]

Julie Houghton[1]

Julian Blake[2]

Anum Shaikh[3]

Edward CF Wilson[3]

Lee Shepstone[4]

Affiliations:

[1]School of Health Sciences, Faculty of Medicine and Health Sciences, University of East Anglia, Norwich, Norfolk, United Kingdom.

[2]Department of Neurophysiology, Norfolk and Norwich University Hospitals NHS foundation Trust, Norwich, UK

[3] Cambridge Centre for Health Services Research, Institute of Public Health, University of Cambridge, Cambridge, UK

[4]Norwich Medical School, Faculty of Medicine and Health Sciences, University of East Anglia, Norwich, Norfolk, United Kingdom.

Corresponding author:

Dr Christina Jerosch-Herold, School of Health Sciences, Faculty of Medicine and Health Sciences, University of East Anglia, Norwich, Norfolk, NR4 7TJ United Kingdom. Email: c.jerosch-herold@uea.ac.uk

Funding:

CJH was funded by the National Institute for Health Research (NIHR Senior Research Fellowship and NIHR Clinical Doctoral Research Fellowship, respectively). ECFW is funded by the NIHR Cambridge Biomedical Research Centre. The funders of the study had no role in study design, data collection, data analysis, data interpretation, or writing of the report. The views and opinions expressed therein are those of the authors and do not necessarily reflect those of the NIHR, NHS or the Department of Health.

Conflict of interest:

We affirm that we have no financial affiliation (including research funding) or involvement with any commercial organization that has a direct financial interest in any matter included in this manuscript, except as disclosed in an attachment and cited in the manuscript.

For peer review only - http://bmjopen.bmj.com/site/about/guidelines.xhtml

For BMJ Open

Acknowledgements:

We thank the patients who participated in this study; the local Principal Investigators at the following collaborating NHS sites: Julian Blake and Adrian Tearle (Norfolk and Norwich University Hospitals NHS Trust), Ann Harvey (Ipswich Hospital), Stephen Scott (James Paget Hospital, Gt Yarmouth), Jeremy Bland (Kings College Hospital), Lionel Christopher Bainbridge (Royal Derby Hospital); lay members from the local Patient and Public Involvement in Research group: Jenny Griffiths and Sue Spooner; Tony Dyer and Antony Colles for database support (Norwich Clinical Trials Unit)

Contributorship:

CJH conceived the PALMS cohort study and obtained funding. CJH, JB, LS and ECFW developed the protocol. JH conducted data collection and data entry. LS, AS and ECFW undertook the statistical analyses. All authors contributed to the drafting of the manuscript and have read and approved the final manuscript.

Data Sharing Statement: no additional data are available

For peer review only

For BMJ Open

Abstract

Objectives:

The PALMS study is designed to identify prognostic factors for outcome from corticosteroid injection and surgical decompression for carpal tunnel syndrome (CTS) and predictors of cost over 2 years. The aim of this paper is to explore the cross-sectional association of baseline patient-reported and clinical severity with anxiety, depression, health-related quality of life and costs of CTS in patients referred to secondary care.

Methods:

Prospective, multi-centre cohort study initiated in 2013. We collected baseline data on patient-reported symptom severity (CTS-6), psychological status (HADS), comorbidities, EQ5D-3L and sociodemographic variables. Nerve conduction tests classified patients into five severity grades (mild to very severe). Data were analysed using a general linear model.

Results:

753 patients with CTS provided complete baseline data. Multivariable linear regression adjusting for age, sex, ethnicity, duration of CTS, smoking status, alcohol consumption, employment status, body mass index and comorbidities showed a highly statistically significant relationship between CTS-6 and anxiety, depression and the EQ-5D ($p<0.0001$ in each case). Likewise, a significant relationship was observed between electrodiagnostic severity and anxiety ($p=0.027$) but not with depression ($p=0.986$) or the EQ-5D ($p=0.257$). NHS and societal costs in the 3 months prior to enrolment were significantly associated with self-reported severity ($p<0.0001$) but not with electrodiagnostic severity.

Conclusions:

Patient-reported symptom severity in carpal tunnel syndrome is significantly and positively associated with anxiety, depression, health-related quality of life and NHS and societal costs even when adjusting for age, gender, body mass index, comorbidities, smoking, drinking and occupational status. In contrast there is little or no evidence of any relationship with objectively derived CTS severity. Further research is needed to understand the possible direction of cause and effect and whether treatment can modify this association.

Article Summary – Strengths and limitations

- Large multi-centre prospective cohort study of CTS (n>700)
- We used a multivariable model to test the independence of associations with a range of pre-specified demographic and clinical variables
- All patients had a diagnosis of CTS made through the combination of clinical history, signs and symptoms with objective electrodiagnostic testing
- Cross-sectional analysis limited to baseline data only with longitudinal (2 year) follow-up still in progress.
- Cost data is limited to a 3 months horizon prior to enrolment

For peer review only - http://bmjopen.bmj.com/site/about/guidelines.xhtml

Introduction

Carpal tunnel syndrome (CTS) is the most common nerve entrapment syndrome; an estimated 1 in 10 people are likely to develop symptoms at some stage in their lives [1]. CTS is characterised by pins and needles, pain and numbness, often affecting both hands. Symptoms can range from intermittent to constant and from very mild to very severe, potentially interfering significantly with daily activities [2]. Whilst in some people CTS resolves spontaneously or responds to conservative treatment by corticosteroid injection, many will require surgical treatment. Surgical decompression rates range from 1/3rd to 2/3rd after initial corticosteroid injection varying by the country and referral criteria [3 4 5].

54,000 carpal tunnel decompressions were carried out in England in 2014-15 (Hospital Episode Statistics) at a cost of £46 million to the NHS (based on an NHS tariff of £864) and are predicted to increase two-fold by 2030 [6]. Clinical Commissioning Groups (CCGs) in England have introduced guidelines on funding for procedures deemed to be of 'limited clinical value', recommending carpal tunnel surgery only for those with moderate to severe CTS and who have not responded to conservative measures (ie corticosteroid injections and splints). A recent review of policies from 175 CCGs in England highlighted that these were highly variable in terms of criteria for defining severity, the necessity for nerve conduction studies (NCS) and duration of conservative treatments before referral for surgery is allowed [7]. Controversy continues over the best management of CTS at different levels of severity and it remains unknown what impact CTS has on patients' mental health, their health-related quality of life, the NHS and society.

The objectives of this study were i) to explore the association of self-reported symptom severity and electrodiagnostic severity with anxiety, depression and health-related quality of life in patients referred for diagnosis and treatment of CTS; and ii) to describe NHS and societal costs of CTS in the 3 months preceding referral for nerve conduction testing and treatment and their association with carpal tunnel severity.

Methods

<u>Design and study population</u>

A prospective longitudinal cohort study was initiated in 2013 to develop a multivariable prognostic model of predictive factors for outcome after steroid injection, outcome after surgery and costs in CTS. The full study protocol has been described elsewhere [8]. Patient recruitment took place at four neurophysiology departments and one hand surgery centre across five NHS Trusts in England.

For peer review only - http://bmjopen.bmj.com/site/about/guidelines.xhtml

For peer review only

Patients with CTS confirmed through a combination of symptoms, clinical history and NCS were invited to participate by returning completed screening questionnaires and signed consent forms. Patients were included if they had CTS in at least one hand confirmed by NCS (grade ≥1) and were aged 18 years or over. Idiopathic CTS as well as CTS secondary to diabetes or hypothyroidism were included. Exclusion criteria were previous surgery in the affected hand in the last 12 months, pregnancy or up to 12-month post-partum, serious comorbidities, other limb mononeuropathies, sensory or motor disturbances secondary to stroke or multiple sclerosis, nerve injury and inability to speak or write English.

For the present study data collected at baseline were used. Self-reported severity and NCS for only one hand per patient was used. In patients with bilateral CTS the 'index hand' was defined as either the worst hand or the dominant hand where self-reported symptom severity was the same bilaterally.

Ethical approval was given by the NRES Committee East of England – Norfolk on 22nd April 2013 (reference 13/EE/0106) and local Research Governance approval at each participating trust was obtained prior to recruitment.

Data collection

A baseline questionnaire was developed comprising the following standardised and non-standardised questionnaires:

- Symptom severity was assessed with the 6-item shortened form of the Boston Carpal Tunnel Questionnaire (BCTQ) [9], the CTS-6 [10] for each hand. The scores range from 1 (no symptoms) to 5 (very severe symptoms).
- Hand functional status was assessed with 3 sub-scales of the Michigan Hand Questionnaire (MHQ) [11 12] : overall hand function for left and right hand, bilateral activities, activities of daily living and work performance. Each subscale is converted into a percentage with higher values denoting greater disability.
- Psychological status was assessed using the Hospital Anxiety and Depression Scale (HADS) [13].
- Health-related quality of life (HRQoL) was assessed by the EuroQoL EQ5D-3L[14].
- Comorbidities were collected by the Self-Administered Comorbidity Questionnaire (SACQ) [15]. The total score ranges from 0 to 36 which is based on 12 listed conditions for which the patient indicates if they have it (1 point), receive treatment for it (2 points) and whether it is activity limiting (3 points).

For peer review only - http://bmjopen.bmj.com/site/about/guidelines.xhtml

- Health care resource use in the 3 months prior to enrolment. This included questions about treatments received (including alternative therapies), medications (prescription and over-the-counter), healthcare contacts (NHS primary, secondary and tertiary, and private), time off work due to CTS and assistance with activities of daily living due to CTS.

Other clinical and demographic variables collected at baseline were age, sex, duration of symptoms, height and weight, work status and type, smoking status, alcohol units consumed per week, ethnicity and household income. Additionally, the NCS reports of enrolled patients were obtained from the participating sites and were graded using Bland's criteria [16] from 1 (mild) to 6 (extremely severe).

Patients were given the option of completing the study questionnaires online via a personalised link to a password protected web-based data entry system maintained by the Norwich Clinical Trials Unit or via paper-based questionnaires sent by mail.

<u>Statistical analysis</u>

A general linear model (GLM) with a normally distributed error term was used to model the HADS anxiety score, HADS depression score and EQ-5D utility score. EQ-5D responses were converted to utilities using UK-specific preference weights [17]. The model included gender, age, ethnicity, employment status, smoking status, units of alcohol per week, body mass index (BMI), duration of CTS and comorbidity score as explanatory variables before adding either CTS-6 symptom score or NCS score. Parameter estimates for the effect of CTS-6 and NCS score are provided with 95% confidence intervals and statistical significance set at the conventional 5% level.

Results

Recruitment took place over 30 months between July 2013 and December 2015. A total of 1918 patients with CTS were identified and invited to participate of which a total of 754 patients met all eligibility criteria and returned full baseline questionnaires (see Strobe diagram in Figure 1).

The sociodemographic and clinical variables of the cohort are summarised in Tables 1 and 2. Bilateral CTS affected 69% of patients and 75% of participants had their symptoms for at least 6 months or longer. Using their worst hand as the index hand only 18% had received a steroid injection and mean self-reported symptom severity (CTS-6) was 2.89 points. Based upon nerve conduction tests, 69% of patients had at least moderate CTS (grades ≥3), with 24% in the severe and very severe categories (grades 5 or 6) (see Table 2).

For peer review only - http://bmjopen.bmj.com/site/about/guidelines.xhtml

The HADS anxiety and HADS depression scores were within normal ranges (score 0 to 7) in 66% and 80% of participants respectively. Self-reported comorbidities are summarised in Table 3. The mean SACQ score was 5.22. The three commonest co-morbidities were low back pain (LBP), osteoarthritis (OA) and high blood pressure with LBP and OA also reported as the most activity limiting. Mean VAS score on the EQ5D-3L was 73.5 (SD= 18.2), and the mean health utility was 0.66. Over 85% of respondents indicated having at least some pain and 31% of the cohort indicated having both pain and feeling anxious or depressed.

Using a multivariable regression model including age, sex, ethnicity, duration of CTS, smoking status, alcohol consumption, employment status, BMI and comorbidity score as potential confounding variables, a highly statistically significant association was found between self-reported symptom severity (using CTS-6 score) and anxiety, depression and HRQoL ($p<0.0001$ in each case). Table 4 gives the parameter estimates for psychological outcome and quality of life by self-reported and objectively graded severity. For each 1 point increase in score on the CTS-6 there was an estimated 1.11 points increase in anxiety score, 1.43 points increase in depression score and a 10% mean decrease in health utility. Patients with the highest symptom severity (4 to 5 points on CTS-6) had mean anxiety scores of 9.62 (SD=5.48) indicating at least mild anxiety (cut off 7 points). There was a marked lower health utility for those with the highest symptom severity (score 4 to 5 on CTS-6) with a mean health utility of 0.43 compared to 0.77 in the mildest group (score 1 to 2 on CTS-6). When including the same independent variables in the model, objective severity grading based on NCS was significantly associated with anxiety ($p=0.027$), but not with depression or HRQoL. The association between NCS grade and anxiety, however, was negative with higher anxiety scores observed in severity grades 1 and 2 and the lowest mean anxiety scores observed in the worst severity group (mean 3.42). For every one grade increase in neurophysiologically assessed severity there was an estimated 0.26 point decrease in the HADS anxiety score.

The mean cost of NHS service use per patient in the three months prior to baseline assessment was £447 (SD= £274), with a societal cost (NHS plus patient out of pocket and lost productivity) of £626 (SD= £655). NHS costs comprised predominantly hospital visits including NCS (£362), GP consultations (£61), prescription medications (£14) and other contacts and treatments (£10), which accounted for 71% of societal costs. Lost productivity (£93) accounted for 15%, and out of pocket costs (£56 for lost wages due to GP/hospital visits, £7 for travel to the hospital, £4 for hand splints and £18 for other contacts and treatments for the remaining 14%. Although those individuals over the age of 40 generally have higher NHS and lower societal costs compared to 18-40 year olds, age is not a significant predictor of costs under either perspective. NHS and societal costs, however,

For peer review only - http://bmjopen.bmj.com/site/about/guidelines.xhtml

significantly increase with self-reported severity of CTS (p<0.0001), but not NCS grade (p=0.928 and p=0.448). For every point increase in the self-reported severity, NHS and societal costs increase by 10% and 20%, respectively (see Table 5).

Discussion

Principal findings:

We found that at the point of referral for NCS and prior to any treatment patients with more severe symptoms reported greater psychological distress and poorer quality of life. Even after adjusting for known and potential confounders such as comorbidities, age, gender and BMI, we observed that every one point increase in symptom severity score was associated with an estimated 1.11 points increase in mean anxiety score, a 1.43 point increase in depression score and a 0.10 decrease in health utility. Adjusting for the same independent variables, objectively graded severity from NCS, however, showed a significant negative association with anxiety and no significant association with depression or health utility.

Average NHS and societal costs per patient were £447 and £626, respectively. There was no significant association between costs, age and objectively graded severity. We found, however, for every point increase in subjective severity, a 10% and 20% relative increase in NHS and societal costs, respectively.

To the best of our knowledge this is the first study to explore the association of self-reported and objectively graded symptom severity with psychological distress and quality of life using a multivariable model whilst adjusting for the effect of other confounders. It is also the first study to measure prospectively the NHS resource use and societal costs of patients with CTS. We included a wide range of variables which could account for increased psychological distress and decreased quality of life including comorbidities weighted by how activity limiting they are, smoking status, alcohol consumption, age, gender, ethnicity, employment status and duration of CTS.

A surprising finding is that objective severity assessed by NCS showed only a weak but negative association with anxiety and there were no significant associations between NCS and HRQoL or NCS and depression. There has been much debate over the value of NCS in the diagnosis of CTS [1 18 19 20] and in the UK not all patients have NCS [7]. Several studies have examined the association of self-

For peer review only - http://bmjopen.bmj.com/site/about/guidelines.xhtml

reported symptoms and function with objective severity from electrodiagnostic findings. Most have used simple correlations with the exception of Chan et al [21]. The latter used a multivariable model controlling for several demographic variables, as well as psychological measures including depression, somatisation and pain-related catastrophizing, in a sample of 215 patients with CTS. They conclude that subjective symptoms and objective severity are independent of each other. One possible explanation is that NCS can only measure the velocity and amplitude of large myelinated fibres that is sensory and motor axons. Yet CTS also affects the small unmyelinated fibres [22] and it is these which account for the neuropathic pain component manifesting as pain during day and night time. Only three out of the six questions in the CTS-6 address sensory symptoms (daytime numbness or tingling, night time numbness or tingling and waking from numbness and tingling) with the other three questions relating to pain at night, in the day and waking due to pain. Patients with more severe electrodiagnostic CTS (grade 5 and 6) have numbness and even muscle wasting but often do not have any 'positive' symptoms such as tingling or pain resulting in a lower overall self-reported symptom score. It has also been suggested that those with more severe compression often have less subjective symptoms, especially in older patients in whom there is a natural age-related decline in sensory function and reduced pain sensitivity [23] but greater functional disability [24]. Another reason for the lack of association with nerve conduction results is that CTS causes small fibre loss not detected by NCS and, surprisingly, in some people with more severe nerve entrapment subjective symptoms and function improve suggesting an adaptive mechanism [22].

Our findings as well as other studies clearly indicate that NCS should not be used as measure of disease impact and neither is it necessary in everyone to make a diagnosis of CTS. However, NCS have an important role in informing treatment planning and have been identified as a strong predictor of treatment response to surgery [25]. For example, in patients with severe and very severe compression (grades 5 or 6) surgery may not reverse the effects of chronic denervation and functional outcome is often poorer as the capacity for recovery is much more limited [25].

Our analysis, being cross-sectional, cannot address the question of whether anxiety and depression are the cause or the effect of self-reported symptom severity. There may well be a bidirectional relationship between symptom severity and psychological distress, whereby each contributes to the development and is a consequence of the other. Neither can we conclude from these data that if symptom severity improves after treatment psychological distress or quality of life would also improve. A possible explanation for the significant independent association between self-reported symptoms, psychological status and HRQoL is that people either consistently underplay or

For peer review only - http://bmjopen.bmj.com/site/about/guidelines.xhtml

exaggerate their symptoms, irrespective of whether these are physical, psychological or global HRQoL. This may also explain the increased costs, both from an NHS and personal perspective, driven by increased treatment-seeking behaviour in those who perceive their symptoms as worse.

Health-related QoL was markedly lower in those with very severe self-reported symptoms (score 4 to 5 on CTS-6). Their mean health utility of 0.43 is much lower than that reported in CTS patients prior to undergoing surgery using the EQ-5D and which range from 0.74 [26] to 0.81 [27]. In fact utility values for this group are closer to those reported by EQ-5D in people with serious diseases like severe heart failure [28] moderate to severe psychotic illness [29] and digestive system cancers [30].

Strengths and limitations:

The strengths of the present study include its prospective design, multi-centre recruitment and a large sample size (>700) in whom a wide range of pre-specified sociodemographic and clinical variables were collected at baseline. Using a multivariable model mitigates the potentially confounding factors such as age, comorbidities, etc. by adjusting for these and thus testing the independence of any associations between self-reported or neurophysiological severity and psychological distress or quality of life.

There are also limitations, namely that the data collection was reliant on patient-self-report which is open to recall bias and data quality could not be independently verified. For example, duration of symptoms may be difficult to attribute separately to each hand in the case of bilateral symptoms or to remember accurately over a longer period. Patients were recruited through secondary care having been referred by a primary care physician. It is therefore possible that our study sample is biased towards those actively seeking treatment for their CTS. Long term follow-up over 2 years is underway and will allow us to explore predictors of treatment related outcomes and cost.

Conclusions

This study shows that even when accounting for potential confounders such as, for example comorbidities or age, a strong and highly significant association remains between symptom severity and psychological status and health-related quality of life. This means that those with more severe symptoms are also more likely to present with worst mental health and poorer quality of life, thus placing a significant burden on the individual, health services and society. Therefore, access to timely and effective diagnostics and treatment before symptoms become severe or very severe is

For peer review only

paramount. Whilst NCS remains a useful tool in the diagnosis of CTS, and provides important information to inform treatment planning, our data suggest that NCS should not be used as a measure of disease impact.

For peer review only

For peer review only - http://bmjopen.bmj.com/site/about/guidelines.xhtml

For BMJ Open

Tables and Figures

Table 1: Demographics

Sex	*Male*	260 (35%)
	Female	493 (65%)
Age (years)	*Mean (SD)*	60.3 (12.7)
Ethnicity	*White*	731 (97%)
	Other	20 (3%)
	Missing	2
BMI	*Mean (SD)*	28.7 (5.8)
Smoking status	*Current*	76 (10%)
	Ex-smoker	299 (40%)
	Non-smoker	375 (50%)
	Missing	3
Alcohol intake (units)	*None*	251 (34%)
	1-4	271 (37%)
	5-10	114 (15%)
	11-20	81 (11%)
	> 21	25 (3%)
	Missing	11
Working status	*Working:*	296 (39%)
	Self-employed	88 (11.7%)
	not working:	457 (61%)
	retired	322 (43%)
	unemployed	14 (1.9%)
	long-term sick	36 (4.8%)
	carer, volunteer or student	85 (11.3%)
Income (per annum)	*< £15K*	170 (23%)
	£15K - £21.5K	103 (14%)
	£21.5 - £35K	148 (20%)
	£35K - £50K	81 (11%)
	> £50K	49 (7%)
	Missing	5

For peer review only

For BMJ Open

Table 2: Clinical Characteristics

Duration of CTS	<3 months	42 (6%)
	3-6 months	140 (19%)
	6-12 months	181 (24%)
	12-18 months	98 (13%)
	>18 months	292 (39%)
Side of CTS	Left	89 (12%)
	Right	148 (20%)
	Bilateral	516 (69%)
Dominant Hand Affected	Yes	669 (89%)
	No	84 (11%)
Worst Side	Left	264 (35%)
	Right	489 (65%)
Worst Side NCS Grade	1	152 (20%)
	2	79 (11%)
	3	202 (27%)
	4	137 (18%)
	5	159 (21%)
	6	24 (3%)
CTS-6 (1 to 5)	Mean (SD)	2.89 (0.85)
MHQ overall function (0-100)	Mean (SD)	53.4 (22.6)
MHQ activity (0-100)	Mean (SD)	66.6 (28.6)
MHQ bilateral activity (0-100)	Mean (SD)	69.4 (25.5)
MHQ work (0-100)	Mean (SD)	64.7 (25.6)
HADS Anxiety	Mean (SD)	6.18 (4.52)
	Normal (1-7)	497 (66%)
	Mild (8-11)	123 (16%)
	Moderate (11-14)	90 (12%)
	Severe (15-21)	42 (6%)
	Missing	1
HADS Depression	Mean (SD)	4.48 (3.84)
	Normal (1-7)	602 (80%)
	Mild (8-10)	88 (12%)
	Moderate (11-14)	47 (6%)
	Severe (15-21)	15 (2%)
	Missing	1
Overall Co-morbidity Score (0-36)	Mean (SD)	5.22 (4.17)
EQ5D-3L VAS	Mean (SD)	73.54 (18.2)

For peer review only

Table 3: Self-reported Comorbidities

Comorbidity	Has problem	Receives treatment	Limits activity
Back pain	342 (45%)	142 (19%)	226 (30%)
Osteoarthritis	253 (34%)	129 (17%)	185 (25%)
High Blood Pressure	235 (31%)	226 (30%)	22 (3%)
Depression	148 (20%)	117 (16%)	63 (8%)
Diabetes	77 (10%)	63 (8%)	11 (1%)
Rheumatoid Arthritis	70 (9%)	40 (5%)	51 (7%)
Lung disease	29 (4%)	23 (3%)	18 (2%)
Cancer	18 (2%)	13 (2%)	5 (<1%)
Kidney disease	14 (2%)	5 (<1%)	3 (<1%)
Liver disease	4 (<1%)	1 (<1%)	0
Ulcer or stomach disease	37 (5%)	32 (4%)	7 (1%)
Other Condition 1	372 (49%)	280 (37%)	150 (20%)
Other Condition 2	140 (19%)	95 (13%)	60 (8%)
Other Condition 3	48 (6%)	30 (4%)	20 (3%)

For peer review only

For BMJ Open

	HADS ANX Mean (SD)	*Estimate (SE)*[1]	*HADS DEP Mean (SD)*	*Estimate (SE)*[1]	*EQ-5D Mean (SD)*	*Estimate (SE)*[1]
CTS-6						
1	3.14 (2.61)		1.57 (1.81)		0.78 (0.32)	
1 – 2	5.04 (4.07)		2.90 (3.12)		0.77 (0.20)	
2 – 3	5.50 (4.05)		3.86 (3.31)		0.72 (0.20)	
3 – 4	6.87 (4.55)		5.42 (3.99)		0.60 (0.28)	
4 – 5	9.62 (5.48)	1.11 (0.19) **p<0.0001**	7.68 (4.44)	1.43 (0.15) **p<0.0001**	0.43 (0.34)	-0.10 (0.01) **p<0.0001**
NCS Grade						
1	6.78 (4.79)		4.64 (4.10)		0.66 (0.28)	
2	7.13 (4.78)		4.78 (3.73)		0.68 (0.25)	
3	6.44 (4.36)		4.33 (3.77)		0.68 (0.27)	
4	6.24 (4.54)		4.80 (3.92)		0.68 (0.26)	
5	5.18 (4.24)		4.20 (3.80)		0.66 (0.24)	
6	3.42 (3.09)	-0.26 (0.18) **p=0.029**	4.08 (3.13)	-0.00 (0.10) p=0.983	0.61 (0.26)	-0.01 (0.01) p=0.214

Table 4: Relationship between CTS Severity and Anxiety, Depression and Quality of Life

1: Parameter estimate (standard error) from a linear model including gender, age, ethnicity, employment status, smoking status, units of alcohol per week, BMI, bilateral CTS and comorbidity score.

For peer review only

For BMJ Open

	NHS cost (SD)	*exp(b)(SE)[1]*	*Societal cost (SD)*	*exp(b)(SE)[1]*
CTS-6				
1	£637.56 (£525.54)		£945.82 (£1,022.42)	
1 – 2	£419.29 (£178.31)		£533.50 (£364.90)	
2 – 3	£419.87 (£214.40)		£552.22 (£493.42)	
3 – 4	£473.48 (£356.10)		£692.13 (£813.95)	
4 – 5	£508.41 (£267.58)	1.10 (0.02) **p<0.0001**	£867.55 (£907.28)	1.20 (0.04) **p<0.0001**
NCS Grade				
1	£460.28 (£439.79)		£591.80 (£547.25)	
2	£465.15 (£289.69)		£632.61 (£523.93)	
3	£447.62 (£242.55)		£664.35 (£759.86)	
4	£415.31 (£155.18)		£533.49 (£255.21)	

Table 5: Relationship between CTS Severity, Age and Cost

For peer review only - http://bmjopen.bmj.com/site/about/guidelines.xhtml

For BMJ Open

5	£447.30 (£215.72)		£680.50 (£868.35)	
6	£482.05 (£233.84)	1.001 (0.02) p=0.928	£597.29 (£286.94)	1.02 (0.03) p=0.448
Age (& CTS-6)				
Age 18 – 40	£433.41 (£178.60)		£758.67 (£1,099.72)	
Age 41 – 60	£444.53 (£226.94)	1.03 (0.09)(p=0.696)	£613.03 (£533.45)	0.83 (0.11)(p=0.175)
Age 61 – 80	£451.34 (£340.08)	1.14 (0.10)(p=0.163)	£603.62 (£637.40)	0.89 (0.14)(p=0.439)
Age 81 plus	£446.70 (£140.83)	1.17 (0.14)(p=0.188)	£745.88 (£952.60)	1.09 (0.22)(p=0.664)
Age (& NCS Grade)				
Age 18 – 40	£433.41 (£178.60)		£758.67 (£1,099.72)	
Age 41 – 60	£444.53 (£226.94)	1.03 (0.09)(p=0.747)	£613.03 (£533.45)	0.82 (0.12)(p=0.176)
Age 61 – 80	£451.34 (£340.08)	1.11 (0.11)(p=0.268)	£603.62 (£637.40)	0.84 (0.14)(p=0.285)
Age 81 plus	£446.70 (£140.83)	1.13 (0.14)(p=0.327)	£745.88 (£952.60)	0.98 (0.21)(p=0.928)

1: Exponentiated parameter estimate (standard error) from a GLM model with log-link and gamma distributed errors including gender, age, ethnicity, employment status, smoking status, units of alcohol per week, BMI, bilateral CTS, co-morbidities and comorbidity score.

For peer review only

For peer review only - http://bmjopen.bmj.com/site/about/guidelines.xhtml

For BMJ Open

References

1. Padua L, Coraci D, Erra C, et al. Carpal tunnel syndrome: clinical features, diagnosis, and management. The Lancet Neurology;**15**(12):1273-84.
2. Bland JDP. Carpal tunnel syndrome. Curr Opin Neurol 2005;**18**(5):581-85.
3. Latinovic R, Gulliford MC, Hughes RAC. Incidence of common compressive neuropathies in primary care. J Neurol Neurosur Ps 2006;**77**(2):263-65.
4. Meys V, Thissen S, Rozeman S, et al. Prognostic factors in carpal tunnel syndrome treated with a corticosteroid injection. Muscle & nerve 2011;**44**(5):763-8.
5. Hameso A, Bland JD. Prevalence of decompression surgery in patients with carpal tunnel syndrome 8 years after initial treatment with a local corticosteroid injection. The Journal of hand surgery, European volume 2017;**42**(3):275-80.
6. Bebbington E, Furniss D. Linear regression analysis of Hospital Episode Statistics predicts a large increase in demand for elective hand surgery in England. J Plast Reconstr Aes 2015;**68**(2):243-51.
7. Ryan D, Shaw A, Graham S, et al. Variation in CCG policies for the treatment of carpal tunnel syndrome. RCS Bulletin 2017;**99**(1):28-31.
8. Jerosch-Herold C, Shepstone L, Wilson EC, et al. Clinical course, costs and predictive factors for response to treatment in carpal tunnel syndrome: the PALMS study protocol. BMC musculoskeletal disorders 2014;**15**:35.
9. Levine D, Simmons B, Koris M, et al. A self-administered questionnaire for the assessment of severity of symptoms and functional status in carpal tunnel syndrome. The Journal of Bone and Joint Surgery 1993;**75A**(11):1585-92.
10. Atroshi I, Lyren PE, Gummesson C. The 6-item CTS symptoms scale: a brief outcomes measure for carpal tunnel syndrome. Qual Life Res 2009;**18**(3):347-58.
11. Chung KC, Pillsbury MS, Walters MR, et al. Reliability and validity testing of the Michigan Hand Outcomes Questionnaire. J Hand Surg-Am 1998;**23A**(4):575-87.
12. Kotsis SV, Chung KC. Responsiveness of the Michigan Hand Outcomes Questionnaire and the Disabilities of the Arm, Shoulder and Hand Questionnaire in carpal tunnel surgery. J Hand Surg-Am 2005;**30A**(1):81-86.
13. Snaith RP, Zigmond AS. The Hospital Anxiety and Depression Scale. Brit Med J 1986;**292**(6516):344-44.
14. Group TE. EuroQol-a new facility for themeaudrment of health-related qulaity of life Health Policy 1990;**16**(3):199-208.
15. Sangha O, Stucki G, Liang MH, et al. The self-administered comorbidity questionnaire: A new method to assess comorbidity for clinical and health services research. Arthrit Rheum-Arthr 2003;**49**(2):156-63.
16. Bland JDP. A neurophysiological grading scale for carpal tunnel syndrome. Muscle & nerve 2000;**23**(8):1280-83.
17. Dolan P. Modeling valuations for EuroQol health states. Med Care 1997;**35**(11):1095-108.
18. Sears ED, Swiatek PR, Hou H, et al. Utilization of Preoperative Electrodiagnostic Studies for Carpal Tunnel Syndrome: An Analysis of National Practice Patterns. J Hand Surg-Am 2016;**41**(6):665-72.
19. Thomson JG. Diagnosis and treatment of carpal tunnel syndrome -correspondence. The Lancet Neurology;**16**(4):263.
20. Padua L, Coraci D, Erra C, et al. Diagnosis and treatment of carpal tunnel syndrome - Authors' response. The Lancet Neurology;**16**(4):263-64.
21. Chan L, Turner JA, Comstock BA, et al. The relationship between electrodiagnostic findings and patient symptoms and function in carpal tunnel syndrome. Archives of physical medicine and rehabilitation 2007;**88**(1):19-24.

For peer review only - http://bmjopen.bmj.com/site/about/guidelines.xhtml

For BMJ Open

22. Schmid AB, Bland JDP, Bhat MA, et al. The relationship of nerve fibre pathology to sensory function in entrapment neuropathy. Brain 2014;**137**:3186-99.
23. Povlsen B, Aggelakis K, Koutroumanidis M. Effect of age on subjective complaints and objective severity of carpal tunnel syndrome: prospective study. JRSM short reports 2010;**1**(7):62.
24. Hobby JL, Venkatesh R, Motkur P. The effect of age and gender upon symptoms and surgical outcomes in carpal tunnel syndrome. J Hand Surg-Brit Eur 2005;**30B**(6):599-604.
25. Bland JDP. Do nerve conduction studies predict the outcome of carpal tunnel decompression? Muscle & nerve 2001;**24**(7):935-40.
26. Nazari G, MacDermid JC, Bain J, et al. Estimation of health-related-quality of life depends on which utility measure is selected for patients with carpal tunnel syndrome. Journal of hand therapy : official journal of the American Society of Hand Therapists 2016.
27. Marti C, Hensler S, Herren DB, et al. Measurement properties of the EuroQoL EQ-5D-5L to assess quality of life in patients undergoing carpal tunnel release. The Journal of hand surgery, European volume 2016.
28. Dyer MT, Goldsmith KA, Sharples LS, et al. A review of health utilities using the EQ-5D in studies of cardiovascular disease. Health and quality of life outcomes 2010;**8**(1):13.
29. Hakkaart L, Lamers LM, Bouwmans CAM, et al. Comparison of EQ-5D and SF-6D in mental health patients. J Ment Health Policy 2007;**10**:S17-S17.
30. Pickard AS, Wilke CT, Lin HW, et al. Health utilities using the EQ-5D in studies of cancer. Pharmacoeconomics 2007;**25**(5):365-84.

For peer review only

Figure 1 : STROBE flowchart [31]

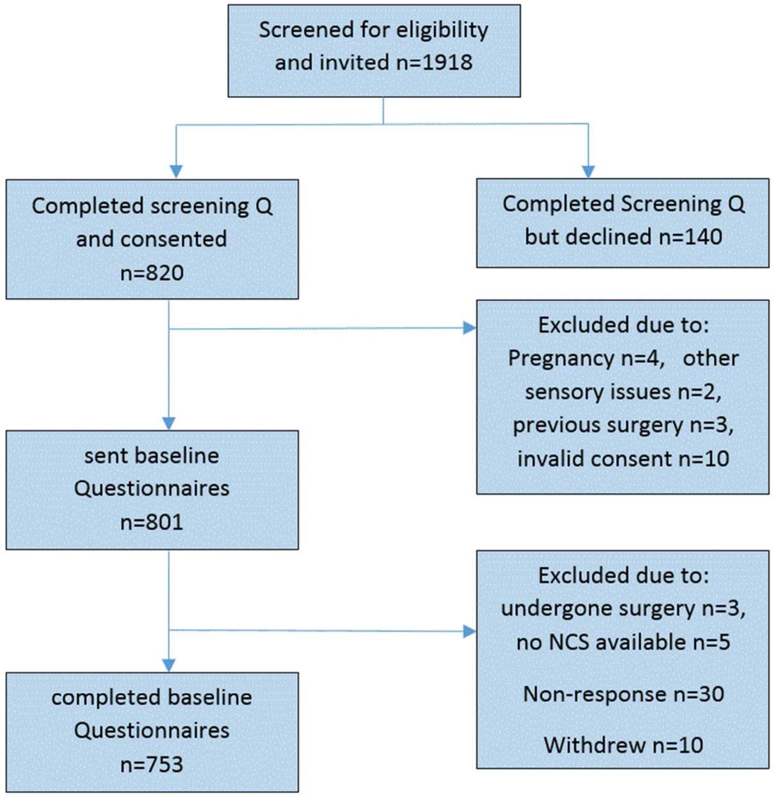

Figure 1: STROBE Flow diagram

331x279mm (72 x 72 DPI)

For peer review only - http://bmjopen.bmj.com/site/about/guidelines.xhtml

STROBE Statement—checklist of items that should be included in reports of observational studies

	Item No	Recommendation	Page
**Title and abstract**	1	(*a*) Indicate the study's design with a commonly used term in the title or the abstract	**3**
		(*b*) Provide in the abstract an informative and balanced summary of what was done and what was found	**3**
**Introduction**			
Background/rationale	2	Explain the scientific background and rationale for the investigation being reported	**4**
Objectives	3	State specific objectives, including any prespecified hypotheses	**4**
**Methods**			
Study design	4	Present key elements of study design early in the paper	**3/4**
Setting	5	Describe the setting, locations, and relevant dates, including periods of recruitment, exposure, follow-up, and data collection	**4/5**
Participants	6	(*a*) *Cohort study*—Give the eligibility criteria, and the sources and methods of selection of participants. Describe methods of follow-up<br>*Case-control study*—Give the eligibility criteria, and the sources and methods of case ascertainment and control selection. Give the rationale for the choice of cases and controls<br>*Cross-sectional study*—Give the eligibility criteria, and the sources and methods of selection of participants	**5**
		(*b*) *Cohort study*—For matched studies, give matching criteria and number of exposed and unexposed<br>*Case-control study*—For matched studies, give matching criteria and the number of controls per case	
Variables	7	Clearly define all outcomes, exposures, predictors, potential confounders, and effect modifiers. Give diagnostic criteria, if applicable	**5/6**
Data sources/ measurement	8*	For each variable of interest, give sources of data and details of methods of assessment (measurement). Describe comparability of assessment methods if there is more than one group	**6**
Bias	9	Describe any efforts to address potential sources of bias	**6**
Study size	10	Explain how the study size was arrived at	**n/a**
Quantitative variables	11	Explain how quantitative variables were handled in the analyses. If applicable, describe which groupings were chosen and why	**6**
Statistical methods	12	(*a*) Describe all statistical methods, including those used to control for confounding	**6**
		(*b*) Describe any methods used to examine subgroups and interactions	**6**
		(*c*) Explain how missing data were addressed	**6**
		(*d*) *Cohort study*—If applicable, explain how loss to follow-up was addressed<br>*Case-control study*—If applicable, explain how matching of cases and controls was addressed<br>*Cross-sectional study*—If applicable, describe analytical methods taking account of sampling strategy	**5/6**
		(*e*) Describe any sensitivity analyses	

For peer review only - http://bmjopen.bmj.com/site/about/guidelines.xhtml

Results			page
Participants	13*	(a) Report numbers of individuals at each stage of study—eg numbers potentially eligible, examined for eligibility, confirmed eligible, included in the study, completing follow-up, and analysed	**6**
		(b) Give reasons for non-participation at each stage	
		(c) Consider use of a flow diagram	**Fig 1**
Descriptive data	14*	(a) Give characteristics of study participants (eg demographic, clinical, social) and information on exposures and potential confounders	**12**
		(b) Indicate number of participants with missing data for each variable of interest	**12**
		(c) *Cohort study*—Summarise follow-up time (eg, average and total amount)	**n/a**
Outcome data	15*	*Cohort study*—Report numbers of outcome events or summary measures over time	
		Case-control study—Report numbers in each exposure category, or summary measures of exposure	
		Cross-sectional study—Report numbers of outcome events or summary measures	**12/13**
Main results	16	(*a*) Give unadjusted estimates and, if applicable, confounder-adjusted estimates and their precision (eg, 95% confidence interval). Make clear which confounders were adjusted for and why they were included	**7, 15, 16**
		(*b*) Report category boundaries when continuous variables were categorized	**n/a**
		(*c*) If relevant, consider translating estimates of relative risk into absolute risk for a meaningful time period	**n/a**
Other analyses	17	Report other analyses done—eg analyses of subgroups and interactions, and sensitivity analyses	
**Discussion**			
Key results	18	Summarise key results with reference to study objectives	**8**
Limitations	19	Discuss limitations of the study, taking into account sources of potential bias or imprecision. Discuss both direction and magnitude of any potential bias	**10**
Interpretation	20	Give a cautious overall interpretation of results considering objectives, limitations, multiplicity of analyses, results from similar studies, and other relevant evidence	**10**
Generalisability	21	Discuss the generalisability (external validity) of the study results	**10**
**Other information**			
Funding	22	Give the source of funding and the role of the funders for the present study and, if applicable, for the original study on which the present article is based	**1**

*Give information separately for cases and controls in case-control studies and, if applicable, for exposed and unexposed groups in cohort and cross-sectional studies.

Note: An Explanation and Elaboration article discusses each checklist item and gives methodological background and published examples of transparent reporting. The STROBE checklist is best used in conjunction with this article (freely available on the Web sites of PLoS Medicine at http://www.plosmedicine.org/, Annals of Internal Medicine at http://www.annals.org/, and Epidemiology at http://www.epidem.com/). Information on the STROBE Initiative is available at www.strobe-statement.org.

For peer review only

For peer review only - http://bmjopen.bmj.com/site/about/guidelines.xhtml

BMJ Open

Association of psychological distress, quality of life and costs with carpal tunnel syndrome severity: a cross-sectional analysis of the PALMS cohort

Journal:	*BMJ Open*
Manuscript ID	bmjopen-2017-017732.R1
Article Type:	Research
Date Submitted by the Author:	13-Jul-2017

Complete List of Authors:	Jerosch-Herold, Christina; University of East Anglia, Health Sciences<br>Houghton, Julie; University of East Anglia Faculty of Medicine and Health Sciences, Health Sciences<br>Blake, Julian; Norfolk and Norwich University Hospitals NHS Foundation Trust, Neurophysiology<br>Shaikh, Anum; University of Cambridge Department of Public Health and Primary Care<br>Wilson, Edward; University of Cambridge, Cambridge Centre for Health Services Research<br>Shepstone, Lee; University of East Anglia Faculty of Medicine and Health Sciences

**Primary Subject Heading**:	Health services research
Secondary Subject Heading:	Health economics, Mental health, General practice / Family practice, Neurology
Keywords:	Neurological pain < NEUROLOGY, Neurophysiology < NEUROLOGY, Hand & wrist < ORTHOPAEDIC & TRAUMA SURGERY, Musculoskeletal disorders < ORTHOPAEDIC & TRAUMA SURGERY, PRIMARY CARE, Depression & mood disorders < PSYCHIATRY

SCHOLARONE™
Manuscripts

Title

Association of psychological distress, quality of life and costs with carpal tunnel syndrome severity: a cross-sectional analysis of the PALMS cohort

Authors

Christina Jerosch-Herold[1]

Julie Houghton[1]

Julian Blake[2]

Anum Shaikh[3]

Edward CF Wilson[3]

Lee Shepstone[4]

Affiliations:

[1]School of Health Sciences, Faculty of Medicine and Health Sciences, University of East Anglia, Norwich, Norfolk, United Kingdom.

[2]Department of Neurophysiology, Norfolk and Norwich University Hospitals NHS foundation Trust, Norwich, UK

[3] Cambridge Centre for Health Services Research, Institute of Public Health, University of Cambridge, Cambridge, UK

[4]Norwich Medical School, Faculty of Medicine and Health Sciences, University of East Anglia, Norwich, Norfolk, United Kingdom.

Corresponding author:

Dr Christina Jerosch-Herold, School of Health Sciences, Faculty of Medicine and Health Sciences, University of East Anglia, Norwich, Norfolk, NR4 7TJ United Kingdom. Email: c.jerosch-herold@uea.ac.uk

Funding:

CJH was funded by the National Institute for Health Research (NIHR) through a NIHR Senior Research Fellowship. ECFW is funded by the NIHR Cambridge Biomedical Research Centre. The funders of the study had no role in study design, data collection, data analysis, data interpretation, or writing of the report. The views and opinions expressed therein are those of the authors and do not necessarily reflect those of the NIHR, NHS or the Department of Health.

Conflict of interest:

We affirm that we have no financial affiliation (including research funding) or involvement with any commercial organization that has a direct financial interest in any matter included in this manuscript, except as disclosed in an attachment and cited in the manuscript.

For peer review only - http://bmjopen.bmj.com/site/about/guidelines.xhtml

For peer review only

Acknowledgements:

We thank the patients who participated in this study; the local Principal Investigators at the following collaborating NHS sites: Julian Blake and Adrian Tearle (Norfolk and Norwich University Hospitals NHS Trust), Ann Harvey (Ipswich Hospital), Stephen Scott (James Paget Hospital, Gt Yarmouth), Jeremy Bland (Kings College Hospital), Lionel Christopher Bainbridge (Royal Derby Hospital); lay members from the local Patient and Public Involvement in Research group: Jenny Griffiths and Sue Spooner; Tony Dyer and Antony Colles for database support (Norwich Clinical Trials Unit)

Contributorship:

CJH conceived the PALMS cohort study and obtained funding. CJH, JB, LS and ECFW developed the protocol. JH conducted data collection and data entry. LS, AS and ECFW undertook the statistical analyses. All authors contributed to the drafting of the manuscript and have read and approved the final manuscript.

Data Sharing Statement: no additional data are available

For peer review only

Abstract

Objectives:

The PALMS study is designed to identify prognostic factors for outcome from corticosteroid injection and surgical decompression for carpal tunnel syndrome (CTS) and predictors of cost over 2 years. The aim of this paper is to explore the cross-sectional association of baseline patient-reported and clinical severity with anxiety, depression, health-related quality of life and costs of CTS in patients referred to secondary care.

Methods:

Prospective, multi-centre cohort study initiated in 2013. We collected baseline data on patient-reported symptom severity (CTS-6), psychological status (HADS), hand function (Michigan Hand Questionnaire) comorbidities, EQ5D-3L and sociodemographic variables. Nerve conduction tests classified patients into five severity grades (mild to very severe). Data were analysed using a general linear model.

Results:

753 patients with CTS provided complete baseline data. Multivariable linear regression adjusting for age, sex, ethnicity, duration of CTS, smoking status, alcohol consumption, employment status, body mass index and comorbidities showed a highly statistically significant relationship between CTS-6 and anxiety, depression and the EQ-5D ($p<0.0001$ in each case). Likewise, a significant relationship was observed between electrodiagnostic severity and anxiety ($p=0.027$) but not with depression ($p=0.986$) or the EQ-5D ($p=0.257$). NHS and societal costs in the 3 months prior to enrolment were significantly associated with self-reported severity ($p<0.0001$) but not with electrodiagnostic severity.

Conclusions:

Patient-reported symptom severity in carpal tunnel syndrome is significantly and positively associated with anxiety, depression, health-related quality of life and NHS and societal costs even when adjusting for age, gender, body mass index, comorbidities, smoking, drinking and occupational status. In contrast there is little or no evidence of any relationship with objectively derived CTS severity. Further research is needed to understand the possible direction of cause and effect and whether treatment can modify this association.

Article Summary – Strengths and limitations

- Large multi-centre prospective cohort study of CTS (n>700)
- We used a multivariable model to test the independence of associations with a range of pre-specified demographic and clinical variables
- All patients had a diagnosis of CTS made through the combination of clinical history, signs and symptoms with objective electrodiagnostic testing
- Cross-sectional analysis limited to baseline data only with longitudinal (2 year) follow-up still in progress.
- Cost data is limited to a 3 months horizon prior to enrolment

For peer review only

Introduction

Carpal tunnel syndrome (CTS) is the most common nerve entrapment syndrome; an estimated 1 in 10 people are likely to develop symptoms at some stage in their lives [1]. CTS is characterised by pins and needles, pain and numbness, often affecting both hands. Symptoms can range from intermittent to constant and from very mild to very severe, potentially interfering significantly with daily activities [2]. Whilst in some people symptoms of CTS resolve spontaneously or respond to non-operative treatment by corticosteroid injection, many undergo surgical decompression to prevent irreversible nerve damage. Decompression rates range from 1/3rd to 2/3rd after initial corticosteroid injection. [3 4 5]. Variations in surgical rates may be due to different follow-up periods in individual studies as well as difference in criteria for and access to surgical decompression.

54,000 carpal tunnel decompressions were carried out in England in 2014-15 (Hospital Episode Statistics) at a cost of £46 million to the NHS (based on an NHS tariff of £864) and are predicted to increase two-fold by 2030 [6]. Clinical Commissioning Groups (CCGs) in England have introduced guidelines on funding for procedures deemed to be of 'limited clinical value', recommending carpal tunnel surgery only for those with '*moderate to severe CTS*' or who have '*not responded to conservative measures'* (ie corticosteroid injections and splints). However it is often unclear whether severity refers to pathophysiology or symptoms or a combination of both. A recent review of policies from 175 CCGs in England highlighted that these were highly variable in terms of criteria for defining severity, the necessity for nerve conduction studies (NCS) and duration of non-operative treatment before referral for surgery is allowed [7]. Controversy continues over the best management of CTS at different stages. More recent research on CTS has highlighted the role of psychological factors such as anxiety, depression and patient's health beliefs in how patients perceive their symptoms, their impact on function and the outcome from treatment [8-10]. No previous studies have explored the association of disease and symptom severity with health-related quality of life or costs from a personal and societal perspective and which may also play an important role in treatment planning.

The objectives of this cross-sectional analysis were i) to explore the association of self-reported symptom severity and electrodiagnostic severity with anxiety, depression, hand function and health-related quality of life in patients referred for diagnosis and treatment of CTS; and ii) to describe NHS and societal costs of CTS in the 3 months preceding referral for nerve conduction testing and treatment and their association with carpal tunnel severity.

For peer review only - http://bmjopen.bmj.com/site/about/guidelines.xhtml

Methods

Design and study population

A prospective longitudinal cohort study (PALMS study) was initiated in 2013 to develop a multivariable prognostic model of predictive factors for outcome after steroid injection, outcome after surgery and costs in CTS. The full study protocol has been described elsewhere [11]. Recruitment took place at five secondary care sites across five NHS Trusts in England where patients had been referred by primary care for nerve conduction tests and prior to any treatment. Patients were diagnosed with CTS by a neurophysiologist or hand surgeon based on signs and symptoms, clinical history and objective measurable pathophysiology (NCS). Eligible patients were invited to participate by returning completed screening questionnaires and signed consent forms. Patients were included if they had CTS in at least one hand confirmed by NCS (grade ≥1) and were aged 18 years or over. NCS grading was done according Bland's criteria[12] (for details see Supplementary Table I). Patients with concomitant diseases such as diabetes or hypothyroidism were included. Exclusion criteria were previous surgery in the affected hand in the last 12 months, pregnancy or up to 12-months post-partum, serious comorbidities, other limb mononeuropathies, sensory or motor disturbances secondary to stroke, multiple sclerosis or nerve injury and inability to speak or write English.

For the present study data collected at baseline were used. Self-reported symptom severity and NCS for only one hand per patient was used. In patients with bilateral CTS the 'index hand' was defined as either the worst hand or the dominant hand where self-reported symptom severity on the CTS-6 was the same bilaterally.

Ethical approval was given by the NRES Committee East of England – Norfolk on 22nd April 2013 (reference 13/EE/0106) and local Research Governance approval at each participating trust was obtained prior to recruitment.

Data collection

A baseline questionnaire was developed comprising the following standardised and non-standardised questionnaires:

- Symptom severity was assessed with the 6-item shortened form of the Boston Carpal Tunnel Questionnaire (BCTQ) [13], the CTS-6 [14] for each hand. The scores range from 1 (no symptoms) to 5 (very severe symptoms).
- Hand functional status was assessed with 3 sub-scales of the Michigan Hand Questionnaire (MHQ) [15 16] : overall hand function for worst hand, unilateral and bilateral activities of daily

For peer review only - http://bmjopen.bmj.com/site/about/guidelines.xhtml

living and work performance. Each subscale is converted into a percentage with higher values denoting greater disability.

- Psychological status was assessed using the Hospital Anxiety and Depression Scale (HADS) [17]. It is made up of 14 items, 7 relating to anxiety and 7 relating to depression. Responses are scored from 0-3 giving a possible score range of 0 to 21 for each subscale. Scores of 8 to 10 identify mild cases, 11 to 15 moderate cases and 16 or more, severe cases. [17]
- Health-related quality of life (HRQoL) was assessed by the EuroQoL EQ5D-3L[18].
- Comorbidities were collected by the Self-Administered Comorbidity Questionnaire (SACQ) [19]. The total score ranges from 0 to 36 which is based on 12 listed conditions for which the patient indicates if they have it (1 point), receive treatment for it (2 points) and whether it is activity limiting (3 points).
- Health care resource use in the 3 months prior to enrolment. This included questions about treatments received (including alternative therapies), medications (prescription and over-the-counter), healthcare contacts (NHS primary, secondary and tertiary, and private), patient-reported days off work due to CTS and assistance with activities of daily living due to CTS.

Other clinical and demographic variables collected at baseline were age, sex, duration of symptoms, height and weight, work status and type, smoking status, alcohol units consumed per week, ethnicity and household income. Additionally, the full NCS reports of enrolled patients were obtained from the participating sites and were graded by the first author using Bland's criteria[12] from 1 (mild) to 6 (extremely severe) (see Supplementary Table I)

Patients were given the option of completing the study questionnaires online via a personalised link to a password protected web-based data entry system maintained by the Norwich Clinical Trials Unit or via paper-based questionnaires sent by mail.

<u>Statistical analysis</u>

A general linear model (GLM) with a normally distributed error term was used to model the HADS anxiety score, HADS depression score, MHQ scores and EQ-5D utility index. EQ-5D responses were converted to utilities using UK-specific preference weights [20]. The model included gender, age, ethnicity, employment status, smoking status, units of alcohol per week, body mass index (BMI), duration of CTS and comorbidity score as explanatory variables before adding either CTS-6 symptom

For peer review only - http://bmjopen.bmj.com/site/about/guidelines.xhtml

score or NCS score. Parameter estimates for the effect of CTS-6 and NCS score are provided with 95% confidence intervals and statistical significance set at the conventional 5% level.

Resource use data were converted to cost per patient using standard UK unit cost sources [Personal Social Services Research Unit (PSSRU), British National Formulary (BNF), and Office for National Statistics (ONS)], the relevant published literature and consultation with experts. Costs were then modelled using a GLM with log link and gamma distributed errors. The base year for the costs was 2015/2016 and the analysis was conducted from the perspectives of the NHS and society (defined as the sum of NHS and patient out-of-pocket costs and morbidity-related lost productivity).

For peer review only

Results

Recruitment took place over 30 months between July 2013 and December 2015. A total of 1918 patients with CTS were identified and invited to participate of which a total of 753 patients met all eligibility criteria and returned full baseline questionnaires (see Strobe diagram in Figure 1).

The sociodemographic and clinical variables of the cohort are summarised in Tables 1 and 2. Bilateral CTS affected 69% of patients and 75% of participants had their symptoms for at least 6 months or longer. Using their worst hand as the index hand only 18% had received a steroid injection and mean self-reported symptom severity (CTS-6) was 2.89 points. Based upon nerve conduction tests, 69% of patients had at least moderate CTS (grades ≥3), with 24% in the severe and very severe categories (grades 5 or 6) (see Table 2).

The mean CTS-6 scores increased steadily with NCS grade, up to grade 6 (which contained relatively few individuals). The mean (and standard deviation) by grade were as follows: grade 1, 2.69 (0.86); grade 2, 2.79 (0.85); grade 3, 2.87 (0.91); grade 4, 3.00 (0.79); grade 5, 3.06 (0.79); grade 6, 2.89 (0.78).

The HADS anxiety and HADS depression scores were within normal ranges (score 0 to 7) in 66% and 80% of participants, respectively. Self-reported comorbidities are summarised in Table 3. The mean SACQ score was 5.22. The three commonest co-morbidities were low back pain (LBP), osteoarthritis (OA) and high blood pressure with LBP and OA also reported as the most activity limiting. Mean VAS score on the EQ5D-3L was 73.5 (SD= 18.2), and the mean health utility index was 0.66. Over 85% of respondents indicated having at least some pain and 31% of the cohort indicated having both pain and feeling anxious or depressed.

Using a multivariable regression model including age, sex, ethnicity, duration of CTS, smoking status, alcohol consumption, employment status, BMI and comorbidity score as potential confounding variables, a highly statistically significant association was found between self-reported symptom

severity (using CTS-6 score) and anxiety, depression and HRQoL (p<0.0001 in each case). Table 4a gives the parameter estimates for psychological outcome and quality of life by self-reported and objectively graded severity. For each 1 point increase in score on the CTS-6 there was an estimated 1.11 points increase in anxiety score, 1.43 points increase in depression score and a 10% mean decrease in health utility. Patients with the highest symptom severity (4 to 5 points on CTS-6) had mean anxiety scores of 9.62 (SD=5.48) indicating at least mild anxiety. There was a marked lower health utility for those with the highest symptom severity (score 4 to 5 on CTS-6) with a mean health utility index of 0.43 compared to 0.77 in the mildest group (score 1 to 2 on CTS-6). When including the same independent variables in the model, objective severity grading based on NCS was significantly associated with anxiety (p=0.027), but not with depression or HRQoL. The association between NCS grade and anxiety, however, was negative with higher anxiety scores observed in severity grades 1 and 2 and the lowest mean anxiety scores observed in the worst severity group (mean 3.42). For every one grade increase in neurophysiologically assessed severity there was an estimated 0.26 point decrease in the HADS anxiety score. Using the same multivariable regression model a highly significant independent association was also found between symptom severity and hand function subscales. For every 1 point increase in symptom severity there was a marked decrease in hand function ranging from 13.4 to 17.8 points on the MHQ (see Table 4b for parameter estimates by subscales). Electrodiagnostic severity was also significantly associated with overall hand function and unilateral and bilateral activities of daily living but not with the work subscale. The magnitude of decline in hand function was less marked when using NCS grade in the model.

The mean cost of NHS service use per patient in the three months prior to baseline assessment was £447 (SD = £274), with a societal cost (NHS plus patient out of pocket and lost productivity) of £636 (SD = £694). NHS costs comprised predominantly hospital visits including NCS (£362), GP consultations (£58), prescription medications (£14) and other contacts and treatments (£13), which accounted for 70% of societal costs. Lost productivity (£101) accounted for 15%, and out of pocket costs (£56 for lost wages due to GP/hospital visits, £7 for travel to the hospital, £4 for hand splints and £18 for other contacts and treatments for the remaining 15%. Although those individuals over the age of 40 generally have higher NHS and lower societal costs compared to 18-40 year olds (reference category), age is not a significant predictor of costs under either perspective. For example, NHS costs are 15% higher on average for those individuals in the 81 plus age group, when compared to 18-40 year olds and accounting for NCS grade, but this, however, is not a significant determinant of costs (p=0.327). NHS and societal costs, however, significantly increase with self-reported severity of CTS (p<0.01 and p<0.0001), but not NCS grade (p=0.269 and p=0.590). For every

For peer review only - http://bmjopen.bmj.com/site/about/guidelines.xhtml

point increase in the self-reported severity, NHS and societal costs increase by 8% and 18%, respectively (see Table 5).

Discussion

Principal findings:

We found that at the point of referral for NCS and prior to any treatment patients with more severe symptoms reported greater psychological distress, poorer hand function and lower quality of life. Even after adjusting for known and potential confounders such as comorbidities, age, gender and BMI, we observed that every one point increase in symptom severity score was associated with an estimated 1.11 points increase in mean anxiety score, a 1.43 point increase in depression score and a 0.10 decrease in health utility. Adjusting for the same independent variables, objectively graded severity from NCS, however, showed a significant negative association with anxiety and no significant association with depression or health utility. When modelling the outcome on self-reported hand function (MHQ) both symptom severity and NCS showed a significant association with overall hand function and activities of daily living, though the decrease in hand function was much more marked when using symptom severity (CTS-6). More than 40% of the variation in hand function was explained by the model when using CTS-6 score and less than 20% when using NCS grade in the model.

Average NHS and societal costs per patient were £447 and £636, respectively. There was no significant association between costs, age and objectively graded severity. We found, however, for every point increase in subjective severity, an 8% and 18% relative increase in NHS and societal costs, respectively.

Several other studies have explored the association of pain severity with a range of psychological variables including anxiety, depression, pain catastrophizing and coping[9 10 21]. These studies all conclude that illness behaviour is a stronger predictor of pain severity than objective measures of disease severity (NCS). In our study we did not measure pain intensity, although the CTS-6 does contain 3 items relating to pain (daytime pain, nocturnal night and waking from pain). It should also be noted that pain is not always the predominant symptom[8].

For peer review only - http://bmjopen.bmj.com/site/about/guidelines.xhtml

To the best of our knowledge this is the first study to explore the association of self-reported and objectively graded symptom severity with psychological distress and quality of life using a multivariable model whilst adjusting for the effect of other confounders. It is also the first study to measure prospectively the NHS resource use and societal costs of patients with CTS. We included a wide range of variables which could account for increased psychological distress and decreased quality of life including comorbidities weighted by how activity limiting they are, smoking status, alcohol consumption, age, gender, ethnicity, employment status and duration of CTS.

A surprising finding is that objective severity assessed by NCS showed only a weak but negative association with anxiety and there were no significant associations between NCS and HRQoL or NCS and depression. There has been much debate over the value of NCS in the diagnosis of CTS [1 22 23 24] and in the UK not all patients have NCS [7]. Several studies have examined the association of self-reported symptoms and function with objective severity from electrodiagnostic findings. Most have used simple correlations with the exception of Chan et al [25]. The latter used a multivariable model controlling for several demographic variables, as well as psychological measures including depression, somatisation and pain-related catastrophizing, in a sample of 215 patients with CTS. They conclude that subjective symptoms and objective severity are independent of each other. One possible explanation is that NCS can only measure the velocity and amplitude of large myelinated fibres that is sensory and motor axons. Yet CTS also affects the small unmyelinated fibres [26] and it is these which account for the neuropathic pain component manifesting as pain during day and night time. Only three out of the six questions in the CTS-6 address sensory symptoms (daytime numbness or tingling, night time numbness or tingling and waking from numbness and tingling) with the other three questions relating to pain at night, in the day and waking due to pain. Patients with more severe electrodiagnostic CTS (grade 5 and 6) have numbness and even muscle wasting but often do not have any ‘positive’ symptoms such as tingling or pain resulting in a lower overall self-reported symptom score. It has also been suggested that those with more severe compression often have less subjective symptoms, especially in older patients in whom there is a natural age-related decline in sensory function and reduced pain sensitivity [27] but greater functional disability [28]. This may also explain the significant association observed between NCS grade and hand function (MHQ) as severe numbness and thenar wasting found in grades 5 and 6 are likely to have a greater impact on hand function.

Another reason for the lack of association with nerve conduction results is that CTS causes small fibre loss not detected by NCS and, surprisingly, in some people with more severe nerve entrapment subjective symptoms and function improve suggesting an adaptive mechanism [26]. Although only

For peer review only - http://bmjopen.bmj.com/site/about/guidelines.xhtml

speculative, it is possible that those with very mild or mild CTS have higher anxiety because they have been misdiagnosed. Another plausible explanation is that in this sample recruited through secondary care, those with very mild CTS represent a more anxious patient group that is also more persistent in seeking medical treatment and referral.

Our findings as well as other studies clearly indicate that NCS should not be used as measure of disease impact and neither is it necessary in everyone to make a diagnosis of CTS. However, NCS have an important role in informing treatment planning and have been identified as a strong predictor of treatment response to surgery [29]. For example, in patients with severe and very severe compression (grades 5 or 6) surgery may not reverse the effects of chronic denervation and functional outcome is often poorer as the capacity for recovery is much more limited [29].

Our analysis, being cross-sectional, cannot address the question of whether anxiety and depression are the cause or the effect of self-reported symptom severity. There may well be a bidirectional relationship between symptom severity and psychological distress, whereby each contributes to the development and is a consequence of the other. Neither can we conclude from these data that if symptom severity improves after treatment then psychological distress or quality of life would also improve. A possible explanation for the significant independent association between self-reported symptoms, psychological status and HRQoL is that people either consistently underplay or exaggerate their symptoms, irrespective of whether these are physical, psychological or global HRQoL. This may also explain the increased costs, both from an NHS and personal perspective, driven by increased treatment-seeking behaviour in those who perceive their symptoms as worse.

Health-related QoL was markedly lower in those with very severe self-reported symptoms (score 4 to 5 on CTS-6). Their mean health utility of 0.43 is much lower than that reported in other studies of CTS patients prior to surgery ranging from 0.74 to 0.81 using the EQ-5D and 0.69 using the SF-36 [30-32]. In fact the mean utility index for this group is closer to that reported by EQ-5D in people with serious diseases like severe heart failure [33] moderate to severe psychotic illness [34] and digestive system cancers [35].

Strengths and limitations:

The strengths of the present study include its prospective design, multi-centre recruitment and a large sample size (>700) in whom a wide range of pre-specified sociodemographic and clinical variables were collected at baseline. Using a multivariable model mitigates the potentially

For peer review only - http://bmjopen.bmj.com/site/about/guidelines.xhtml

confounding factors such as age, comorbidities, etc. by adjusting for these and thus testing the independence of any associations between self-reported or neurophysiological severity and psychological distress, hand function or quality of life.

There are also limitations, namely that the data collection was reliant on patient-self-report which is open to recall bias and data quality could not be independently verified. For example, duration of symptoms may be difficult to attribute separately to each hand in the case of bilateral symptoms or to remember accurately over a longer period. Patients were recruited through secondary care having been referred by a primary care physician. It is therefore possible that our study sample is biased towards those actively seeking treatment for their CTS. Long term follow-up over 2 years is underway and will allow us to explore predictors of treatment related outcomes and cost. We were also not able to explain why costs were higher for the lowest and highest CTS-6 grades, although it should be borne in mind that these costs relate only to the three months prior to entry in the study. The 2 year follow-up data will provide a more robust analysis. Finally our sample is biased towards an older age group and may not be representative of the typical clinical population with CTS.

Conclusions

This study shows that even when accounting for potential confounders such as, for example comorbidities or age, a strong and highly significant association remains between symptom severity and psychological status and health-related quality of life. This means that those with more severe symptoms are also more likely to present with worst mental health, poorer hand function and lower quality of life, thus placing a significant burden on the individual, health services and society. Therefore, access to timely and effective diagnostics and treatment before symptoms become severe or very severe is paramount. Whilst NCS remains a useful tool in the diagnosis of CTS, and provides important information to inform what treatment should be offered, i.e. non-operative or operative and likely prognosis, NCS should not be used as a measure of disease impact.

For peer review only - http://bmjopen.bmj.com/site/about/guidelines.xhtml

For peer review only

Figure Legends Figure 1: STROBE Flowchart

Tables

Table 1: Demographics

Sex	*Male*	260 (35%)
	Female	493 (65%)
Age (years)	*Mean (SD)*	60.3 (12.7)
Ethnicity	*White*	731 (97%)
	Other	20 (3%)
	Missing	2
BMI	*Mean (SD)*	28.7 (5.8)
Smoking status	*Current*	76 (10%)
	Ex-smoker	299 (40%)
	Non-smoker	375 (50%)
	Missing	3
Alcohol intake (units)	*None*	251 (34%)
	1-4	271 (37%)
	5-10	114 (15%)
	11-20	81 (11%)
	> 21	25 (3%)
	Missing	11
Working status	*Working:*	296 (39%)
	Self-employed	88 (11.7%)
	not working:	457 (61%)
	retired	322 (43%)
	unemployed	14 (1.9%)
	long-term sick	36 (4.8%)
	carer, volunteer or student	85 (11.3%)
Income (per annum)	*< £15K*	170 (23%)
	£15K - £21.5K	103 (14%)
	£21.5 - £35K	148 (20%)
	£35K - £50K	81 (11%)
	> £50K	49 (7%)
	Missing	5

For peer review only

Table 2: Clinical Characteristics

Duration of CTS	<3 months	42 (6%)
	3-6 months	140 (19%)
	6-12 months	181 (24%)
	12-18 months	98 (13%)
	>18 months	292 (39%)
Side of CTS	Left	89 (12%)
	Right	148 (20%)
	Bilateral	516 (69%)
Dominant Hand Affected	Yes	669 (89%)
	No	84 (11%)
Worst Side	Left	264 (35%)
	Right	489 (65%)
Worst Side NCS Grade	1	150 (20%)
	2	79 (11%)
	3	202 (27%)
	4	137 (18%)
	5	159 (21%)
	6	24 (3%)
	missing	2
CTS-6 (1 to 5)	Mean (SD)	2.89 (0.85)
MHQ overall function (0-100)	Mean (SD)	53.4 (22.6)
MHQ unilateral activities (0-100)	Mean (SD)	66.6 (28.6)
MHQ bilateral activities (0-100)	Mean (SD)	69.4 (25.5)
MHQ work (0-100)	Mean (SD)	64.7 (25.6)
HADS Anxiety	Mean (SD)	6.18 (4.52)
	Normal (1-7)	497 (66%)
	Mild (8-11)	123 (16%)
	Moderate (11-14)	90 (12%)
	Severe (15-21)	42 (6%)
	Missing	1
HADS Depression	Mean (SD)	4.48 (3.84)
	Normal (1-7)	602 (80%)
	Mild (8-10)	88 (12%)
	Moderate (11-14)	47 (6%)
	Severe (15-21)	15 (2%)
	Missing	1
Overall Co-morbidity Score (0-36)	Mean (SD)	5.22 (4.17)
EQ5D-3L VAS	Mean (SD)	73.54 (18.2)

For peer review only

For peer review only - http://bmjopen.bmj.com/site/about/guidelines.xhtml

Table 3: Self-reported Comorbidities

Comorbidity	Has problem	Receives treatment	Limits activity
Back pain	342 (45%)	142 (19%)	226 (30%)
Osteoarthritis	253 (34%)	129 (17%)	185 (25%)
High Blood Pressure	235 (31%)	226 (30%)	22 (3%)
Depression	148 (20%)	117 (16%)	63 (8%)
Diabetes	77 (10%)	63 (8%)	11 (1%)
Rheumatoid Arthritis	70 (9%)	40 (5%)	51 (7%)
Lung disease	29 (4%)	23 (3%)	18 (2%)
Cancer	18 (2%)	13 (2%)	5 (<1%)
Kidney disease	14 (2%)	5 (<1%)	3 (<1%)
Liver disease	4 (<1%)	1 (<1%)	0
Ulcer or stomach disease	37 (5%)	32 (4%)	7 (1%)
Other Condition 1	372 (49%)	280 (37%)	150 (20%)
Other Condition 2	140 (19%)	95 (13%)	60 (8%)
Other Condition 3	48 (6%)	30 (4%)	20 (3%)

For peer review only

	n	HADS ANX Mean (SD)	Estimate (95% CI)[1]	HADS DEP Mean (SD)	Estimate (95% CI)[1]	EQ-5D Mean (SD)	Estimate (95% CI)[1]
CTS 6 Score							
0 – 1.00	7	3.14 (2.61)		1.57 (1.81)		0.78 (0.32)	
1.01 – 2.00	134	5.04 (4.07)		2.90 (3.12)		0.77 (0.20)	
2.01 – 3.00	308	5.50 (4.05)		3.86 (3.31)		0.72 (0.20)	
3.01 – 4.00	241	6.87 (4.55)		5.42 (3.99)		0.60 (0.28)	
4.01 – 5.00	63	9.62 (5.48)	1.11 (0.73, 1.49) P<0.0001 R^2 = 17.7%	7.68 (4.44)	1.43 (1.13, 1.73) P<0.0001 R^2 = 26.1%	0.43 (0.34)	-0.10 (-0.12, -0.08) P<0.0001 R^2 = 37.4%
NCS Grade							
1	150	6.78 (4.79)		4.64 (4.10)		0.66 (0.28)	
2	79	7.13 (4.78)		4.78 (3.73)		0.68 (0.25)	
3	202	6.44 (4.36)		4.33 (3.77)		0.68 (0.27)	
4	137	6.24 (4.54)		4.80 (3.92)		0.68 (0.26)	
5	159	5.18 (4.24)		4.20 (3.80)		0.66 (0.24)	
6	24	3.42 (3.09)	-0.26 (-0.49, -0.03) P=0.029 R^2 = 14.3%	4.08 (3.13)	-0.00 (-0.20, 0.20) P=0.983 R^2 = 17.1%	0.61 (0.26)	-0.01 (-0.03, 0.01) P=0.214 R^2 = 28.4%

Table 4(a) Relationship between CTS Severity and Anxiety, Depression and Quality of Life

1: Parameter estimate (95% confidence interval) from a linear model including gender, age, ethnicity, employment status, smoking status, units of alcohol per week, BMI, duration of CTS and comorbidity score.

For peer review only

For peer review only - http://bmjopen.bmj.com/site/about/guidelines.xhtml

Table 4(b) Relationship between CTS Severity and MHQ sub-scales

	n	Overall Function Mean (SD)	Estimate (95% CI)[1]	ADL unilateral Mean (SD)	Estimate (95% CI)[1]	ADL bilateral Mean (SD)	Estimate (95% CI)[1]	Work Mean (SD)	Estimate (95% CI)[1]
CTS 6 Score									
0 – 1.00	7	92.1 (10.7)		91.4 (16.5)		88.8 (11.4)		92.9 (12.9)	
1.01 – 2.00	134	68.5 (20.3)		82.9 (22.0)		82.9 (18.9)		78.7 (22.3)	
2.01 – 3.00	308	58.3 (19.1)		74.5 (23.1)		76.2 (20.7)		70.7 (22.8)	
3.01 – 4.00	241	44.9 (18.9)		56.8 (26.3)		60.7 (24.9)		55.0 (23.2)	
4.01 – 5.00	63	25.8 (16.2)	-14.7 (-16.4, -13.1) P<0.0001 R^2 = 41.1%	28.2 (26.4)	-17.8 (-19.9, -15.8) P<0.0001 R^2 = 40.7%	38.3 (26.2)	-14.6 (-16.4, -12.8) P<0.0001 R^2 = 40.8%	39.6 (22.2)	-13.4 (-15.4, -11.5) P<0.0001 R^2 = 33.3%
NCS Grade									
1	150	58.9 (23.9)		71.0 (27.9)		72.7 (24.4)		63.9 (27.4)	
2	79	53.5 (22.9)		71.5 (27.7)		74.1 (25.2)		67.5 (26.2)	
3	202	54.6 (22.8)		70.5 (27.7)		72.6 (24.6)		65.8 (26.0)	
4	137	53.1 (20.5)		67.0 (26.3)		68.2 (24.3)		63.4 (23.9)	
5	159	48.8 (21.2)		57.8 (30.0)		63.5 (27.3)		64.4 (24.4)	
6	24	43.3 (24.9)	-3.16 (-3.56, -1.22) P<0.0001 R^2 = 15.5%	47.9 (29.8)	-3.87 (-5.32, -2.42) P<0.0001 R^2 = 18.7%	52.5 (26.0)	-3.16 (-4.42, -1.90) P<0.0001 R^2 = 21.9%	61.9 (23.6)	-0.86 (-2.18,0.47) P=0.197 R^2 = 15.7%

[1] Parameter estimate (95% confidence interval) from a linear model including gender, age, ethnicity, employment status, smoking status, units of alcohol per week, BMI, duration of CTS and comorbidity score. ADL: activities of daily living

For peer review only - http://bmjopen.bmj.com/site/about/guidelines.xhtml

	n	NHS cost (SD)	exp(b)(95% CI)[1]	Societal cost (SD)	exp(b)(95% CI)[1]
CTS-6					
0-1.00	7	£512.83 (£405.61)		£726.02 (£776.80)	
1.01-2.00	134	£434.04 (£212.53)		£559.37 (£430.53)	
2.01-3.00	308	£422.57 (£220.30)		£554.73 (£492.25)	
3.01-4.00	241	£466.50 (£348.74)		£686.11 (£814.25)	
4.01-5.00	63	£511.52 (£273.65)	1.08 (1.03, 1.12)	£984.19 (£1,197.94)	1.18 (1.10, 1.27)

Table 5: Relationship between CTS Severity, Age and Cost

For peer review only - http://bmjopen.bmj.com/site/about/guidelines.xhtml

			p<0.01		p<0.0001
NCS Grade					
1	150	£483.36 (£456.12)		£629.69 (£608.90)	
2	79	£459.44 (£243.93)		£592.70 (£381.55)	
3	202	£438.50 (£213.31)		£657.58 (£755.06)	
4	137	£414.14 (£153.80)		£578.23 (£597.95)	
5	159	£439.38 (£203.02)		£676.24 (£892.99)	
6	24	£467.71 (£237.77)	0.98 (0.96, 1.01) p=0.269	£583.77 (£294.98)	1.01 (0.96, 1.07) P=0.590
Age & CTS-6					
Age 18 – 40	43	£433.41 (£178.60)		£758.67 (£1,099.72)	
Age 41 – 60	344	£444.53 (£226.94)	1.04 (0.88, 1.22) p=0.679	£613.03 (£533.45)	0.83 (0.63, 1.10) p=0.196
Age 61 – 80	322	£451.96 (£336.62)	1.13 (0.94, 1.36) p=0.190	£631.64 (£731.18)	0.90 (0.66, 1.22) p=0.486
Age 81 plus	44	£442.46 (£146.20)	1.16 (0.91, 1.47) p=0.224	£712.66 (£973.69)	1.09 (0.73, 1.62) p=0.690
Age & NCS Grade					
Age 18 – 40	43	£433.41 (£178.60)		£758.67 (£1,099.72)	
Age 41 – 60	343	£443.81 (£226.88)	1.02 (0.87, 1.21) p=0.774	£608.42 (£527.83)	0.81 (0.60, 1.09) p=0.163
Age 61 – 80	321	£451.29 (£336.93)	1.11 (0.93, 1.34) p=0.246	£629.87 (£730.63)	0.85 (0.61, 1.18) p=0.336
Age 81 plus	44	£442.46 (£146.20)	1.15 (0.90, 1.47) p=0.252	£712.65 (£973.69)	0.99 (0.64, 1.53) p=0.976

[1] Exponentiated parameter estimate (standard error) from a GLM model with log-link and gamma distributed errors including gender, age, ethnicity, employment status, smoking status, units of alcohol per week, BMI, bilateral CTS, co-morbidities and comorbidity score.

For peer review only - http://bmjopen.bmj.com/site/about/guidelines.xhtml

References

1. Padua L, Coraci D, Erra C, et al. Carpal tunnel syndrome: clinical features, diagnosis, and management. Lancet Neurol;**15**(12):1273-84.
2. Bland JDP. Carpal tunnel syndrome. Curr Opin Neurol 2005;**18**(5):581-85.
3. Latinovic R, Gulliford MC, Hughes RAC. Incidence of common compressive neuropathies in primary care. J Neurol Neurosur Psych 2006;**77**(2):263-65.
4. Meys V, Thissen S, Rozeman S, et al. Prognostic factors in carpal tunnel syndrome treated with a corticosteroid injection. Muscle & Nerve 2011;**44**(5):763-8.
5. Hameso A, Bland JD. Prevalence of decompression surgery in patients with carpal tunnel syndrome 8 years after initial treatment with a local corticosteroid injection. J Hand Surg, European Volume 2017;**42**(3):275-80.
6. Bebbington E, Furniss D. Linear regression analysis of Hospital Episode Statistics predicts a large increase in demand for elective hand surgery in England. J Plast Reconstr Aes 2015;**68**(2):243-51.
7. Ryan D, Shaw A, Graham S, et al. Variation in CCG policies for the treatment of carpal tunnel syndrome. RCS Bulletin 2017;**99**(1):28-31.
8. Duckworth AD, Jenkins PJ, Roddam P, et al. Pain and carpal tunnel syndrome. J Hand Surg 2013;**38**(8):1540-6.
9. Nunez F, Vranceanu AM, Ring D. Determinants of pain in patients with carpal tunnel syndrome. Clin OrthRel Res 2010;**468**(12):3328-32.
10. Fernandez-de-las-Penas C, Fernandez-Munoz JJ, Palacios-Cena M, et al. Direct and Indirect Effects of Function in Associated Variables Such as Depression and Severity on Pain Intensity in Women with Carpal Tunnel Syndrome. Pain Medicine 2015;**16**(12):2405-11.
11. Jerosch-Herold C, Shepstone L, Wilson EC, et al. Clinical course, costs and predictive factors for response to treatment in carpal tunnel syndrome: the PALMS study protocol. BMC Musc Dis 2014;**15**:35.
12. Bland JDP. A neurophysiological grading scale for carpal tunnel syndrome. Muscle & Nerve 2000;**23**(8):1280-83.
13. Levine D, Simmons B, Koris M, et al. A self-administered questionnaire for the assessment of severity of symptoms and functional status in carpal tunnel syndrome. J Bone Joint Surg Am 1993;**75A**(11):1585-92.
14. Atroshi I, Lyren PE, Gummesson C. The 6-item CTS symptoms scale: a brief outcomes measure for carpal tunnel syndrome. Qual Life Res 2009;**18**(3):347-58.
15. Chung KC, Pillsbury MS, Walters MR, et al. Reliability and validity testing of the Michigan Hand Outcomes Questionnaire. J Hand Surg-Am 1998;**23A**(4):575-87.
16. Kotsis SV, Chung KC. Responsiveness of the Michigan Hand Outcomes Questionnaire and the Disabilities of the Arm, Shoulder and Hand Questionnaire in carpal tunnel surgery. J Hand Surg-Am 2005;**30A**(1):81-86.
17. Snaith RP, Zigmond AS. The Hospital Anxiety and Depression Scale. Brit Med J 1986;**292**(6516):344-44.
18. EuroQol Group. EuroQol-a new facility for the measurement of health-related quality of life. Health Policy 1990;**16**(3):199-208.
19. Sangha O, Stucki G, Liang MH, et al. The self-administered comorbidity questionnaire: A new method to assess comorbidity for clinical and health services research. Arthrit Rheum-Arthr 2003;**49**(2):156-63.
20. Dolan P. Modeling valuations for EuroQol health states. Med Care 1997;**35**(11):1095-108.
21. Hobby JL, Venkatesh R, Motkur P. The effect of psychological disturbance on symptoms, self-reported disability and surgical outcome in carpal tunnel syndrome. J Bone Joint Surg Br 2005;**87B**(2):196-200.

For peer review only - http://bmjopen.bmj.com/site/about/guidelines.xhtml

22. Sears ED, Swiatek PR, Hou H, et al. Utilization of Preoperative Electrodiagnostic Studies for Carpal Tunnel Syndrome: An Analysis of National Practice Patterns. J Hand Surg-Am 2016;**41**(6):665-72.
23. Thomson JG. Diagnosis and treatment of carpal tunnel syndrome - correspondence. Lancet Neurol;**16**(4):263.
24. Padua L, Coraci D, Erra C, et al. Diagnosis and treatment of carpal tunnel syndrome - Authors' response. Lancet Neurol;**16**(4):263-64.
25. Chan L, Turner JA, Comstock BA, et al. The relationship between electrodiagnostic findings and patient symptoms and function in carpal tunnel syndrome. Arch Phys Med Rehab 2007;**88**(1):19-24.
26. Schmid AB, Bland JDP, Bhat MA, et al. The relationship of nerve fibre pathology to sensory function in entrapment neuropathy. Brain 2014;**137**:3186-99.
27. Povlsen B, Aggelakis K, Koutroumanidis M. Effect of age on subjective complaints and objective severity of carpal tunnel syndrome: prospective study. JRSM Short Reports 2010;**1**(7):62.
28. Hobby JL, Venkatesh R, Motkur P. The effect of age and gender upon symptoms and surgical outcomes in carpal tunnel syndrome. J Hand Surg-Brit Eur 2005;**30B**(6):599-604.
29. Bland JDP. Do nerve conduction studies predict the outcome of carpal tunnel decompression? Muscle & Nerve 2001;**24**(7):935-40.
30. Nazari G, MacDermid JC, Bain J, et al. Estimation of health-related-quality of life depends on which utility measure is selected for patients with carpal tunnel syndrome. J Hand Ther published online December 14, 2016:1-7.
31. Marti C, Hensler S, Herren DB, et al. Measurement properties of the EuroQoL EQ-5D-5L to assess quality of life in patients undergoing carpal tunnel release. J Hand Surg, Eur 2016; **41**(9):957-962.
32. Atroshi I, Gummesson C, McCabe SJ, et al. The SF-6D health utility index in carpal tunnel syndrome. J Hand Surg, Europ Vol 2007;**32**(2):198-202.
33. Dyer MT, Goldsmith KA, Sharples LS, et al. A review of health utilities using the EQ-5D in studies of cardiovascular disease. Health Qual of Life Outcomes 2010;**8**(1):13.
34. Hakkaart L, Lamers LM, Bouwmans CAM, et al. Comparison of EQ-5D and SF-6D in mental health patients. J Ment Health Policy 2007;**10**:S17-S17.
35. Pickard AS, Wilke CT, Lin HW, et al. Health utilities using the EQ-5D in studies of cancer. Pharmacoeconomics 2007;**25**(5):365-84.

For peer review only

For peer review only - http://bmjopen.bmj.com/site/about/guidelines.xhtml

Figure 1 : STROBE flowchart [31]

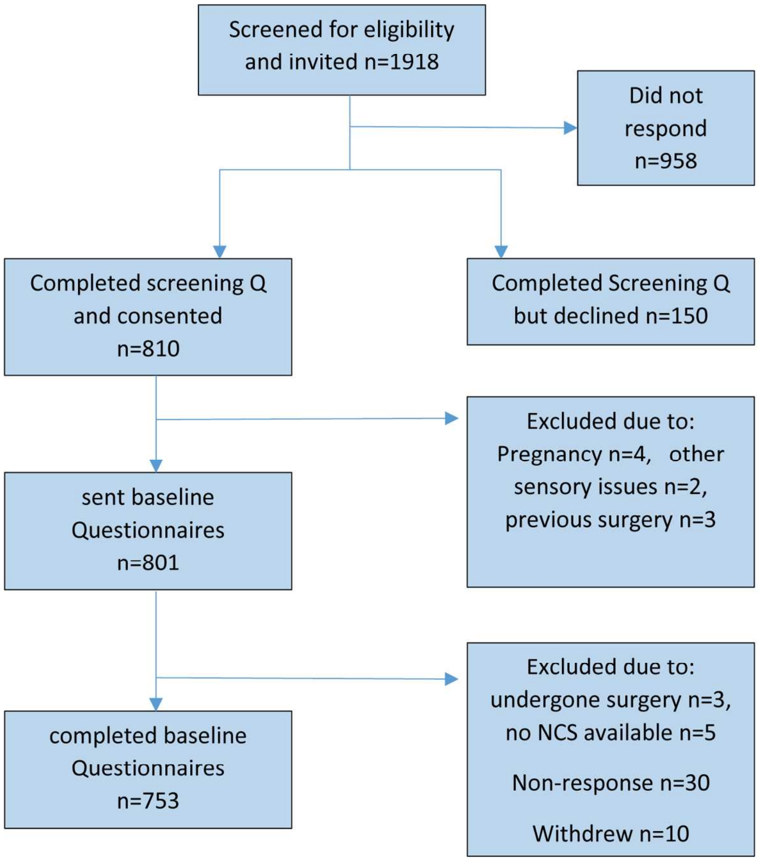

Figure1: STROBE flowchart

156x142mm (300 x 300 DPI)

Supplementary File I:

Criteria for grading of nerve conduction studies:

Grade 0	Normal	No neurophysiological abnormality
Grade 1	Very mild	Median SAP digit II or III to wrist: < 50m/s and ≥40 m/s, Ulnar SAP>50m/s<br>Median DML APB to wrist: >4.0ms and Ulnar DML <=3.8ms<br>or<br>2 sensitive tests positive (ring finger 'double peak' SAP or palm/wrist – median/ulnar comparison or Lumbrical to interosseous latency increase)
Grade 2	Mild	Median SAP digit II or III to wrist SAP: < 40 m/sec<br>Median DML APB to wrist: < 4.5msec
Grade 3	Moderate	Median SAP digit II or III to wrist: present<br>Median DML APB to wrist: > 4.5msec and < 6.5 ms
Grade 4	Severe	Median SAP digit II or III to wrist: absent<br>Median DML APB to wrist: > 4.5 and < 6.5 ms
Grade 5	Very severe	Median DML APB to wrist : > 6.5 msec
Grade 6	Extremely severe	Median DML APB to wrist: unrecordable (< 0.2 mV peak to peak)

SAP: sensory action potential, DML: distal motor latency; APB: Abductor Pollicis Brevis; ms: milliseconds

For peer review only

For peer review only

STROBE Statement—checklist of items that should be included in reports of observational studies

	Item No	Recommendation	Page
**Title and abstract**	1	(*a*) Indicate the study's design with a commonly used term in the title or the abstract	**3**
		(*b*) Provide in the abstract an informative and balanced summary of what was done and what was found	**3**
**Introduction**			
Background/rationale	2	Explain the scientific background and rationale for the investigation being reported	**4**
Objectives	3	State specific objectives, including any prespecified hypotheses	**4**
**Methods**			
Study design	4	Present key elements of study design early in the paper	**3/4**
Setting	5	Describe the setting, locations, and relevant dates, including periods of recruitment, exposure, follow-up, and data collection	**4/5**
Participants	6	(*a*) *Cohort study*—Give the eligibility criteria, and the sources and methods of selection of participants. Describe methods of follow-up<br>*Case-control study*—Give the eligibility criteria, and the sources and methods of case ascertainment and control selection. Give the rationale for the choice of cases and controls<br>*Cross-sectional study*—Give the eligibility criteria, and the sources and methods of selection of participants	**5**
		(*b*) *Cohort study*—For matched studies, give matching criteria and number of exposed and unexposed<br>*Case-control study*—For matched studies, give matching criteria and the number of controls per case	
Variables	7	Clearly define all outcomes, exposures, predictors, potential confounders, and effect modifiers. Give diagnostic criteria, if applicable	**5/6**
Data sources/ measurement	8*	For each variable of interest, give sources of data and details of methods of assessment (measurement). Describe comparability of assessment methods if there is more than one group	**6**
Bias	9	Describe any efforts to address potential sources of bias	**6**
Study size	10	Explain how the study size was arrived at	**n/a**
Quantitative variables	11	Explain how quantitative variables were handled in the analyses. If applicable, describe which groupings were chosen and why	**6**
Statistical methods	12	(*a*) Describe all statistical methods, including those used to control for confounding	**6**
		(*b*) Describe any methods used to examine subgroups and interactions	**6**
		(*c*) Explain how missing data were addressed	**6**
		(*d*) *Cohort study*—If applicable, explain how loss to follow-up was addressed<br>*Case-control study*—If applicable, explain how matching of cases and controls was addressed<br>*Cross-sectional study*—If applicable, describe analytical methods taking account of sampling strategy	**5/6**
		(*e*) Describe any sensitivity analyses	

For peer review only - http://bmjopen.bmj.com/site/about/guidelines.xhtml
For peer review only

**Results**			**page**
Participants	13*	(a) Report numbers of individuals at each stage of study—eg numbers potentially eligible, examined for eligibility, confirmed eligible, included in the study, completing follow-up, and analysed	**6**
		(b) Give reasons for non-participation at each stage	
		(c) Consider use of a flow diagram	**Fig 1**
Descriptive data	14*	(a) Give characteristics of study participants (eg demographic, clinical, social) and information on exposures and potential confounders	**12**
		(b) Indicate number of participants with missing data for each variable of interest	**12**
		(c) *Cohort study*—Summarise follow-up time (eg, average and total amount)	**n/a**
Outcome data	15*	*Cohort study*—Report numbers of outcome events or summary measures over time	
		Case-control study—Report numbers in each exposure category, or summary measures of exposure	
		Cross-sectional study—Report numbers of outcome events or summary measures	**12/13**
Main results	16	(*a*) Give unadjusted estimates and, if applicable, confounder-adjusted estimates and their precision (eg, 95% confidence interval). Make clear which confounders were adjusted for and why they were included	**7, 15, 16**
		(*b*) Report category boundaries when continuous variables were categorized	**n/a**
		(*c*) If relevant, consider translating estimates of relative risk into absolute risk for a meaningful time period	**n/a**
Other analyses	17	Report other analyses done—eg analyses of subgroups and interactions, and sensitivity analyses	
**Discussion**			
Key results	18	Summarise key results with reference to study objectives	**8**
Limitations	19	Discuss limitations of the study, taking into account sources of potential bias or imprecision. Discuss both direction and magnitude of any potential bias	**10**
Interpretation	20	Give a cautious overall interpretation of results considering objectives, limitations, multiplicity of analyses, results from similar studies, and other relevant evidence	**10**
Generalisability	21	Discuss the generalisability (external validity) of the study results	**10**
**Other information**			
Funding	22	Give the source of funding and the role of the funders for the present study and, if applicable, for the original study on which the present article is based	**1**

*Give information separately for cases and controls in case-control studies and, if applicable, for exposed and unexposed groups in cohort and cross-sectional studies.

Note: An Explanation and Elaboration article discusses each checklist item and gives methodological background and published examples of transparent reporting. The STROBE checklist is best used in conjunction with this article (freely available on the Web sites of PLoS Medicine at http://www.plosmedicine.org/, Annals of Internal Medicine at http://www.annals.org/, and Epidemiology at http://www.epidem.com/). Information on the STROBE Initiative is available at www.strobe-statement.org.

For peer review only

For peer review only - http://bmjopen.bmj.com/site/about/guidelines.xhtml

BMJ Open

Association of psychological distress, quality of life and costs with carpal tunnel syndrome severity: a cross-sectional analysis of the PALMS cohort

Journal:	*BMJ Open*
Manuscript ID	bmjopen-2017-017732.R2
Article Type:	Research
Date Submitted by the Author:	14-Aug-2017
Complete List of Authors:	Jerosch-Herold, Christina; University of East Anglia, Health Sciences<br>Houghton, Julie; University of East Anglia Faculty of Medicine and Health Sciences, Health Sciences<br>Blake, Julian; Norfolk and Norwich University Hospitals NHS Foundation Trust, Neurophysiology<br>Shaikh, Anum; University of Cambridge Department of Public Health and Primary Care<br>Wilson, Edward; University of Cambridge, Cambridge Centre for Health Services Research<br>Shepstone, Lee; University of East Anglia Faculty of Medicine and Health Sciences
**Primary Subject Heading**:	Health services research
Secondary Subject Heading:	Health economics, Mental health, General practice / Family practice, Neurology
Keywords:	Neurological pain < NEUROLOGY, Neurophysiology < NEUROLOGY, Hand & wrist < ORTHOPAEDIC & TRAUMA SURGERY, Musculoskeletal disorders < ORTHOPAEDIC & TRAUMA SURGERY, PRIMARY CARE, Depression & mood disorders < PSYCHIATRY

SCHOLARONE™
Manuscripts

For BMJ Open

Title

Association of psychological distress, quality of life and costs with carpal tunnel syndrome severity: a cross-sectional analysis of the PALMS cohort

Authors

Christina Jerosch-Herold[1]

Julie Houghton[1]

Julian Blake[2, 5]

Anum Shaikh[3]

Edward CF Wilson[3]

Lee Shepstone[4]

Affiliations:

[1]School of Health Sciences, Faculty of Medicine and Health Sciences, University of East Anglia, Norwich, Norfolk, United Kingdom.

[2]Department of Clinical Neurophysiology, Norfolk and Norwich University Hospitals NHS foundation Trust, Norwich, UK

[3] Cambridge Centre for Health Services Research, Institute of Public Health, University of Cambridge, Cambridge, UK

[4]Norwich Medical School, Faculty of Medicine and Health Sciences, University of East Anglia, Norwich, Norfolk, United Kingdom.

[5]MRC Centre for Neuromuscular Diseases, Queen Square, London,

Corresponding author:

Dr Christina Jerosch-Herold, School of Health Sciences, Faculty of Medicine and Health Sciences, University of East Anglia, Norwich, Norfolk, NR4 7TJ United Kingdom. Email: c.jerosch-herold@uea.ac.uk

Funding:

CJH was funded by the National Institute for Health Research (NIHR) through a NIHR Senior Research Fellowship. ECFW is funded by the NIHR Cambridge Biomedical Research Centre. The funders of the study had no role in study design, data collection, data analysis, data interpretation, or writing of the report. The views and opinions expressed therein are those of the authors and do not necessarily reflect those of the NIHR, NHS or the Department of Health.

Conflict of interest:

We affirm that we have no financial affiliation (including research funding) or involvement with any commercial organization that has a direct financial interest in any matter included in this manuscript, except as disclosed in an attachment and cited in the manuscript.

For peer review only - http://bmjopen.bmj.com/site/about/guidelines.xhtml

Acknowledgements:

We thank the patients who participated in this study; the local Principal Investigators at the following collaborating NHS sites: Julian Blake and Adrian Tearle (Norfolk and Norwich University Hospitals NHS Trust), Ann Harvey (Ipswich Hospital), Stephen Scott (James Paget Hospital, Gt Yarmouth), Jeremy Bland (Kings College Hospital), Lionel Christopher Bainbridge (Royal Derby Hospital); lay members from the local Patient and Public Involvement in Research group: Jenny Griffiths and Sue Spooner; Tony Dyer and Antony Colles for database support (Norwich Clinical Trials Unit)

Contributorship:

CJH conceived the PALMS cohort study and obtained funding. CJH, JB, LS and ECFW developed the protocol. JH conducted data collection and data entry. LS, AS and ECFW undertook the statistical analyses. All authors contributed to the drafting of the manuscript and have read and approved the final manuscript.

Data Sharing Statement: no additional data are available

For peer review only

For BMJ Open

Abstract

Objectives:

The PALMS study is designed to identify prognostic factors for outcome from corticosteroid injection and surgical decompression for carpal tunnel syndrome (CTS) and predictors of cost over 2 years. The aim of this paper is to explore the cross-sectional association of baseline patient-reported and clinical severity with anxiety, depression, health-related quality of life and costs of CTS in patients referred to secondary care.

Methods:

Prospective, multi-centre cohort study initiated in 2013. We collected baseline data on patient-reported symptom severity (CTS-6), psychological status (HADS), hand function (Michigan Hand Questionnaire) comorbidities, EQ5D-3L and sociodemographic variables. Nerve conduction tests classified patients into five severity grades (mild to very severe). Data were analysed using a general linear model.

Results:

753 patients with CTS provided complete baseline data. Multivariable linear regression adjusting for age, sex, ethnicity, duration of CTS, smoking status, alcohol consumption, employment status, body mass index and comorbidities showed a highly statistically significant relationship between CTS-6 and anxiety, depression and the EQ-5D ($p<0.0001$ in each case). Likewise, a significant relationship was observed between electrodiagnostic severity and anxiety ($p=0.027$) but not with depression ($p=0.986$) or the EQ-5D ($p=0.257$). NHS and societal costs in the 3 months prior to enrolment were significantly associated with self-reported severity ($p<0.0001$) but not with electrodiagnostic severity.

Conclusions:

Patient-reported symptom severity in carpal tunnel syndrome is significantly and positively associated with anxiety, depression, health-related quality of life and NHS and societal costs even when adjusting for age, gender, body mass index, comorbidities, smoking, drinking and occupational status. In contrast there is little or no evidence of any relationship with objectively derived CTS severity. Further research is needed to understand the possible direction of cause and effect and whether treatment can modify this association.

Article Summary – Strengths and limitations

- Large multi-centre prospective cohort study of CTS (n>700)
- We used a multivariable model to test the independence of associations with a range of pre-specified demographic and clinical variables
- All patients had a diagnosis of CTS made through the combination of clinical history, signs and symptoms with objective electrodiagnostic testing
- Cross-sectional analysis limited to baseline data only with longitudinal (2 year) follow-up still in progress.
- Cost data is limited to a 3 months horizon prior to enrolment

For peer review only - http://bmjopen.bmj.com/site/about/guidelines.xhtml

Introduction

Carpal tunnel syndrome (CTS) is the most common nerve entrapment syndrome; an estimated 1 in 10 people are likely to develop symptoms at some stage in their lives [1]. CTS is characterised by pins and needles, pain and numbness, often affecting both hands. Symptoms can range from intermittent to constant and from very mild to very severe, potentially interfering significantly with daily activities[2]. Approximately 1/3rd to 2/3rd of patients with CTS go on to have surgery [3 4 5]. Such variations in surgical rates may be due to different follow-up periods in individual studies as well as differences in criteria for and access to surgical decompression.

54,000 carpal tunnel decompressions were carried out in England in 2014-15 (Hospital Episode Statistics) at a cost of £46 million to the NHS (based on an NHS tariff of £864) and are predicted to increase two-fold by 2030 [6]. Clinical Commissioning Groups (CCGs) in England have introduced guidelines on funding for procedures deemed to be of 'limited clinical value', recommending carpal tunnel surgery only for those with '*moderate to severe CTS*' or who have '*not responded to conservative measures*' (ie corticosteroid injections and splints). However it is often unclear whether severity refers to pathophysiology or symptoms or a combination of both. A recent review of policies from 175 CCGs in England highlighted that these were highly variable in terms of criteria for defining severity, the necessity for nerve conduction studies (NCS) and duration of non-operative treatment before referral for surgery is allowed [7]. Controversy continues over the best management of CTS at different stages. More recent research on CTS has highlighted the role of psychological factors such as anxiety, depression and patient's health beliefs in how patients perceive their symptoms, their impact on function and the outcome from treatment [8-10]. No previous studies have explored the association of disease and symptom severity with health-related quality of life or costs from a personal and societal perspective and which may also play an important role in treatment planning.

The objectives of this cross-sectional analysis were i) to explore the association of self-reported symptom severity and electrodiagnostic severity with anxiety, depression, hand function and health-related quality of life in patients referred for diagnosis and treatment of CTS; and ii) to describe NHS and societal costs of CTS in the 3 months preceding referral for nerve conduction testing and treatment and their association with carpal tunnel severity.

For peer review only - http://bmjopen.bmj.com/site/about/guidelines.xhtml

For BMJ Open

Methods

Design and study population

A prospective longitudinal cohort study (PALMS study) was initiated in 2013 to develop a multivariable prognostic model of predictive factors for outcome after steroid injection, outcome after surgery and costs in CTS. The full study protocol has been described elsewhere [11]. Recruitment took place at five secondary care sites across five NHS Trusts in England where patients had been referred by primary care for nerve conduction tests and prior to any treatment. Patients were diagnosed with CTS by a neurophysiologist or hand surgeon based on signs and symptoms, clinical history and objective measurable pathophysiology (NCS). Eligible patients were invited to participate by returning completed screening questionnaires and signed consent forms. Patients were included if they had CTS in at least one hand confirmed by NCS (grade ≥1) and were aged 18 years or over. NCS grading was done according Bland's criteria[12] (for details see Supplementary Table I). Patients with concomitant diseases such as diabetes or hypothyroidism were included. Exclusion criteria were previous surgery in the affected hand in the last 12 months, pregnancy or up to 12-months post-partum, serious comorbidities, other limb mononeuropathies, sensory or motor disturbances secondary to stroke, multiple sclerosis or nerve injury and inability to speak or write English.

For the present study data collected at baseline were used. Self-reported symptom severity and NCS for only one hand per patient was used. In patients with bilateral CTS the 'index hand' was defined as either the worst hand or the dominant hand where self-reported symptom severity on the CTS-6 was the same bilaterally.

Ethical approval was given by the NRES Committee East of England – Norfolk on 22nd April 2013 (reference 13/EE/0106) and local Research Governance approval at each participating trust was obtained prior to recruitment.

Data collection

A baseline questionnaire was developed comprising the following standardised and non-standardised questionnaires:

- Symptom severity was assessed with the 6-item shortened form of the Boston Carpal Tunnel Questionnaire (BCTQ) [13], the CTS-6 [14] for each hand. The scores range from 1 (no symptoms) to 5 (very severe symptoms). A mean score of 1 indicates no symptoms (tingling, numbness and pain during the past 2 weeks).

For peer review only - http://bmjopen.bmj.com/site/about/guidelines.xhtml

- Hand functional status was assessed with 3 sub-scales of the Michigan Hand Questionnaire (MHQ) [15 16] : overall hand function for worst hand, unilateral and bilateral activities of daily living and work performance. Each subscale is converted into a percentage with higher values denoting greater disability.
- Psychological status was assessed using the Hospital Anxiety and Depression Scale (HADS) [17]. It is made up of 14 items, 7 relating to anxiety and 7 relating to depression. Responses are scored from 0-3 giving a possible score range of 0 to 21 for each subscale. Scores of 8 to 10 identify mild cases, 11 to 15 moderate cases and 16 or more, severe cases. [17]
- Health-related quality of life (HRQoL) was assessed by the EuroQoL EQ5D-3L[18].
- Comorbidities were collected by the Self-Administered Comorbidity Questionnaire (SACQ) [19]. The total score ranges from 0 to 36 which is based on 12 listed conditions for which the patient indicates if they have it (1 point), receive treatment for it (2 points) and whether it is activity limiting (3 points).
- Health care resource use in the 3 months prior to enrolment. This included questions about treatments received (including alternative therapies), medications (prescription and over-the-counter), healthcare contacts (NHS primary, secondary and tertiary, and private), patient-reported days off work due to CTS and assistance with activities of daily living due to CTS.

Other clinical and demographic variables collected at baseline were age, sex, duration of symptoms, height and weight, work status and type, smoking status, alcohol units consumed per week, ethnicity and household income. Additionally, the full NCS reports of enrolled patients were obtained from the participating sites and were graded by the first author using Bland's criteria[12] from 1 (mild) to 6 (extremely severe) (see Supplementary Table I)

Patients were given the option of completing the study questionnaires online via a personalised link to a password protected web-based data entry system maintained by the Norwich Clinical Trials Unit or via paper-based questionnaires sent by mail.

Statistical analysis

A general linear model (GLM) with a normally distributed error term was used to model the HADS anxiety score, HADS depression score, MHQ scores and EQ-5D utility index. EQ-5D responses were converted to utilities using UK-specific preference weights [20]. The model included gender, age, ethnicity, employment status, smoking status, units of alcohol per week, body mass index (BMI),

For peer review only - http://bmjopen.bmj.com/site/about/guidelines.xhtml

duration of CTS and comorbidity score as explanatory variables before adding either CTS-6 symptom score or NCS score. Parameter estimates for the effect of CTS-6 and NCS score are provided with 95% confidence intervals and statistical significance set at the conventional 5% level.

Resource use data were converted to cost per patient using standard UK unit cost sources [Personal Social Services Research Unit (PSSRU), British National Formulary (BNF), and Office for National Statistics (ONS)], the relevant published literature and consultation with experts. Costs were then modelled using a GLM with log link and gamma distributed errors. The base year for the costs was 2015/2016 and the analysis was conducted from the perspectives of the NHS and society (defined as the sum of NHS and patient out-of-pocket costs and morbidity-related lost productivity).

Results

Recruitment took place over 30 months between July 2013 and December 2015. A total of 1918 patients with CTS were identified and invited to participate of which a total of 753 patients met all eligibility criteria and returned full baseline questionnaires (see Strobe diagram in Figure 1).

The sociodemographic and clinical variables of the cohort are summarised in Tables 1 and 2. Bilateral CTS affected 69% of patients and 75% of participants had their symptoms for at least 6 months or longer. Using their worst hand as the index hand only 18% had received a steroid injection and mean self-reported symptom severity (CTS-6) was 2.89 points. Based upon nerve conduction tests, 69% of patients had at least moderate CTS (grades ≥3), with 24% in the severe and very severe categories (grades 5 or 6) (see Table 2).

The mean CTS-6 scores increased steadily with NCS grade, up to grade 6 (which contained relatively few individuals). The mean (and standard deviation) by grade were as follows: grade 1, 2.69 (0.86); grade 2, 2.79 (0.85); grade 3, 2.87 (0.91); grade 4, 3.00 (0.79); grade 5, 3.06 (0.79); grade 6, 2.89 (0.78).

The HADS anxiety and HADS depression scores were within normal ranges (score 0 to 7) in 66% and 80% of participants, respectively. Self-reported comorbidities are summarised in Table 3. The mean SACQ score was 5.22. The three commonest co-morbidities were low back pain (LBP), osteoarthritis (OA) and high blood pressure with LBP and OA also reported as the most activity limiting. Mean VAS score on the EQ5D-3L was 73.5 (SD= 18.2), and the mean health utility index was 0.66. Over 85% of respondents indicated having at least some pain and 31% of the cohort indicated having both pain and feeling anxious or depressed.

For peer review only - http://bmjopen.bmj.com/site/about/guidelines.xhtml

For peer review only

For BMJ Open

Using a multivariable regression model including age, sex, ethnicity, duration of CTS, smoking status, alcohol consumption, employment status, BMI and comorbidity score as potential confounding variables, a highly statistically significant association was found between self-reported symptom severity (using CTS-6 score) and anxiety, depression and HRQoL ($p<0.0001$ in each case). Table 4a gives the parameter estimates for psychological outcome and quality of life by self-reported and objectively graded severity. For each 1 point increase in score on the CTS-6 there was an estimated 1.11 points increase in anxiety score, 1.43 points increase in depression score and a 10% mean decrease in health utility. Patients with the highest symptom severity (4 to 5 points on CTS-6) had mean anxiety scores of 9.62 (SD=5.48) indicating at least mild anxiety. There was a marked lower health utility for those with the highest symptom severity (score 4 to 5 on CTS-6) with a mean health utility index of 0.43 compared to 0.77 in the mildest group (score 1 to 2 on CTS-6). When including the same independent variables in the model, objective severity grading based on NCS was significantly associated with anxiety ($p=0.027$), but not with depression or HRQoL. The association between NCS grade and anxiety, however, was negative with higher anxiety scores observed in severity grades 1 and 2 and the lowest mean anxiety scores observed in the worst severity group (mean 3.42). For every one grade increase in neurophysiologically assessed severity there was an estimated 0.26 point decrease in the HADS anxiety score. Using the same multivariable regression model a highly significant independent association was also found between symptom severity and hand function subscales. For every 1 point increase in symptom severity there was a marked decrease in hand function ranging from 13.4 to 17.8 points on the MHQ (see Table 4b for parameter estimates by subscales). Electrodiagnostic severity was also significantly associated with overall hand function and unilateral and bilateral activities of daily living but not with the work subscale. The magnitude of decline in hand function was less marked when using NCS grade in the model.

The mean cost of NHS service use per patient in the three months prior to baseline assessment was £447 (SD = £274), with a societal cost (NHS plus patient out of pocket and lost productivity) of £636 (SD = £694). NHS costs comprised predominantly hospital visits including NCS (£362), GP consultations (£58), prescription medications (£14) and other contacts and treatments (£13), which accounted for 70% of societal costs. Lost productivity (£101) accounted for 15%, and out of pocket costs (£56 for lost wages due to GP/hospital visits, £7 for travel to the hospital, £4 for hand splints and £18 for other contacts and treatments for the remaining 15%. Although those individuals over the age of 40 generally have higher NHS and lower societal costs compared to 18-40 year olds (reference category), age is not a significant predictor of costs under either perspective. For example, NHS costs are 15% higher on average for those individuals in the 81 plus age group, when

For peer review only - http://bmjopen.bmj.com/site/about/guidelines.xhtml

compared to 18-40 year olds and accounting for NCS grade, but this, however, is not a significant determinant of costs (p=0.327). NHS and societal costs, however, significantly increase with self-reported severity of CTS (p<0.01 and p<0.0001), but not NCS grade (p=0.269 and p=0.590). For every point increase in the self-reported severity, NHS and societal costs increase by 8% and 18%, respectively (see Table 5).

Discussion

Principal findings:

We found that at the point of referral for NCS and prior to any treatment patients with more severe symptoms reported greater psychological distress, poorer hand function and lower quality of life. Even after adjusting for known and potential confounders such as comorbidities, age, gender and BMI, we observed that every one point increase in symptom severity score was associated with an estimated 1.11 points increase in mean anxiety score, a 1.43 point increase in depression score and a 0.10 decrease in health utility. Adjusting for the same independent variables, objectively graded severity from NCS, however, showed a significant negative association with anxiety and no significant association with depression or health utility. When modelling the outcome on self-reported hand function (MHQ) both symptom severity and NCS showed a significant association with overall hand function and activities of daily living, though the decrease in hand function was much more marked when using symptom severity (CTS-6). More than 40% of the variation in hand function was explained by the model when using CTS-6 score and less than 20% when using NCS grade in the model.

Average NHS and societal costs per patient were £447 and £636, respectively. There was no significant association between costs, age and objectively graded severity. We found, however, for every point increase in subjective severity, an 8% and 18% relative increase in NHS and societal costs, respectively.

Several other studies have explored the association of pain severity with a range of psychological variables including anxiety, depression, pain catastrophizing and coping[9 10 21]. These studies all conclude that illness behaviour is a stronger predictor of pain severity than objective measures of disease severity (NCS). In our study we did not measure pain intensity, although the CTS-6 does

For peer review only - http://bmjopen.bmj.com/site/about/guidelines.xhtml

For peer review only

contain 3 items relating to pain (daytime pain, nocturnal night and waking from pain). It should also be noted that pain is not always the predominant symptom[8].

To the best of our knowledge this is the first study to explore the association of self-reported and objectively graded symptom severity with psychological distress and quality of life using a multivariable model whilst adjusting for the effect of other confounders. It is also the first study to measure prospectively the NHS resource use and societal costs of patients with CTS. We included a wide range of variables which could account for increased psychological distress and decreased quality of life including comorbidities weighted by how activity limiting they are, smoking status, alcohol consumption, age, gender, ethnicity, employment status and duration of CTS.

A surprising finding is that objective severity assessed by NCS showed only a weak but negative association with anxiety and there were no significant associations between NCS and HRQoL or NCS and depression. There has been much debate over the value of NCS in the diagnosis of CTS [1 22 23 24] and in the UK not all patients have NCS [7]. Several studies have examined the association of self-reported symptoms and function with objective severity from electrodiagnostic findings. Most have used simple correlations with the exception of Chan et al [25]. The latter used a multivariable model controlling for several demographic variables, as well as psychological measures including depression, somatisation and pain-related catastrophizing, in a sample of 215 patients with CTS. They conclude that subjective symptoms and objective severity are independent of each other. One possible explanation is that NCS can only measure the velocity and amplitude of large myelinated fibres that is sensory and motor axons. Yet CTS also affects the small unmyelinated fibres [26] and it is these which account for the neuropathic pain component manifesting as pain during day and night time. Only three out of the six questions in the CTS-6 address sensory symptoms (daytime numbness or tingling, night time numbness or tingling and waking from numbness and tingling) with the other three questions relating to pain at night, in the day and waking due to pain. Patients with more severe electrodiagnostic CTS (grade 5 and 6) have numbness and even muscle wasting but often do not have any 'positive' symptoms such as tingling or pain resulting in a lower overall self-reported symptom score. It has also been suggested that those with more severe compression often have less subjective symptoms, especially in older patients in whom there is a natural age-related decline in sensory function and reduced pain sensitivity [27] but greater functional disability [28]. This may also explain the significant association observed between NCS grade and hand function (MHQ) as severe numbness and thenar wasting found in grades 5 and 6 are likely to have a greater impact on hand function.

For peer review only - http://bmjopen.bmj.com/site/about/guidelines.xhtml

For BMJ Open

Another reason for the lack of association with nerve conduction results is that CTS causes small fibre loss not detected by NCS and, surprisingly, in some people with more severe nerve entrapment subjective symptoms and function improve suggesting an adaptive mechanism [26]. Although only speculative, it is possible that those with very mild or mild CTS have higher anxiety because they have been misdiagnosed. Another plausible explanation is that in this sample recruited through secondary care, those with very mild CTS represent a more anxious patient group that is also more persistent in seeking medical treatment and referral.

Our findings as well as other studies clearly indicate that NCS should not be used as measure of disease impact and neither is it necessary in everyone to make a diagnosis of CTS. However, NCS have an important role in informing treatment planning and have been identified as a strong predictor of treatment response to surgery [29]. For example, in patients with severe and very severe compression (grades 5 or 6) surgery may not reverse the effects of chronic denervation and functional outcome is often poorer as the capacity for recovery is much more limited [29].

Our analysis, being cross-sectional, cannot address the question of whether anxiety and depression are the cause or the effect of self-reported symptom severity. There may well be a bidirectional relationship between symptom severity and psychological distress, whereby each contributes to the development and is a consequence of the other. Neither can we conclude from these data that if symptom severity improves after treatment then psychological distress or quality of life would also improve. A possible explanation for the significant independent association between self-reported symptoms, psychological status and HRQoL is that people either consistently underplay or exaggerate their symptoms, irrespective of whether these are physical, psychological or global HRQoL. This may also explain the increased costs, both from an NHS and personal perspective, driven by increased treatment-seeking behaviour in those who perceive their symptoms as worse.

Health-related QoL was markedly lower in those with very severe self-reported symptoms (score 4 to 5 on CTS-6). Their mean health utility of 0.43 is much lower than that reported in other studies of CTS patients prior to surgery ranging from 0.74 to 0.81 using the EQ-5D and 0.69 using the SF-36 or SF-6D[30-32]. In fact the mean utility index for this group is closer to that reported by EQ-5D in people with serious diseases like severe heart failure [33] moderate to severe psychotic illness [34] and digestive system cancers [35].

<u>Strengths and limitations:</u>

For peer review only - http://bmjopen.bmj.com/site/about/guidelines.xhtml

The strengths of the present study include its prospective design, multi-centre recruitment and a large sample size (>700) in whom a wide range of pre-specified sociodemographic and clinical variables were collected at baseline. Using a multivariable model mitigates the potentially confounding factors such as age, comorbidities, etc. by adjusting for these and thus testing the independence of any associations between self-reported or neurophysiological severity and psychological distress, hand function or quality of life.

There are also limitations, namely that the data collection was reliant on patient-self-report which is open to recall bias and data quality could not be independently verified. For example, duration of symptoms may be difficult to attribute separately to each hand in the case of bilateral symptoms or to remember accurately over a longer period. Patients were recruited through secondary care having been referred by a primary care physician. It is therefore possible that our study sample is biased towards those actively seeking treatment for their CTS. Long term follow-up over 2 years is underway and will allow us to explore predictors of treatment related outcomes and cost. We were also not able to explain why costs were higher for the lowest and highest CTS-6 grades, although it should be borne in mind that these costs relate only to the three months prior to entry in the study. The 2 year follow-up data will provide a more robust analysis. Finally our sample is biased towards an older age group and may not be representative of the typical clinical population with CTS.

Conclusions

This study shows that even when accounting for potential confounders such as, for example comorbidities or age, a strong and highly significant association remains between symptom severity and psychological status and health-related quality of life. This means that those with more severe symptoms are also more likely to present with worst mental health, poorer hand function and lower quality of life, thus placing a significant burden on the individual, health services and society. Therefore, access to timely and effective diagnostics and effective treatment before symptoms become severe or very severe is paramount. NCS remains a useful tool to assess disease severity, which in turn should be used to determine whether surgery is indicated or not, however NCS should not be used as a measure of the impact of CTS on the person. Patients presenting with only mild disease severity on NCS but who exhibit anxiety or depression may need to be offered additional psychological support.

For peer review only - http://bmjopen.bmj.com/site/about/guidelines.xhtml

For BMJ Open

Tables and Figures

Table 1: Demographics

Sex	*Male*	260 (35%)
	Female	493 (65%)
Age (years)	*Mean (SD)*	60.3 (12.7)
Ethnicity	*White*	731 (97%)
	Other	20 (3%)
	Missing	2
BMI	*Mean (SD)*	28.7 (5.8)
Smoking status	*Current*	76 (10%)
	Ex-smoker	299 (40%)
	Non-smoker	375 (50%)
	Missing	3
Alcohol intake (units)	*None*	251 (34%)
	1-4	271 (37%)
	5-10	114 (15%)
	11-20	81 (11%)
	> 21	25 (3%)
	Missing	11
Working status	*Working:*	296 (39%)
	Self-employed	88 (11.7%)
	not working:	457 (61%)
	retired	322 (43%)
	unemployed	14 (1.9%)
	long-term sick	36 (4.8%)
	carer, volunteer or student	85 (11.3%)
Income (per annum)	*< £15K*	170 (23%)
	£15K - £21.5K	103 (14%)
	£21.5 - £35K	148 (20%)
	£35K - £50K	81 (11%)
	> £50K	49 (7%)
	Missing	5

For peer review only

For peer review only - http://bmjopen.bmj.com/site/about/guidelines.xhtml

Table 2: Clinical Characteristics

Duration of CTS	<3 months	42 (6%)
	3-6 months	140 (19%)
	6-12 months	181 (24%)
	12-18 months	98 (13%)
	>18 months	292 (39%)
Side of CTS	Left	89 (12%)
	Right	148 (20%)
	Bilateral	516 (69%)
Dominant Hand Affected	Yes	669 (89%)
	No	84 (11%)
Worst Side	Left	264 (35%)
	Right	489 (65%)
Worst Side NCS Grade	1	150 (20%)
	2	79 (11%)
	3	202 (27%)
	4	137 (18%)
	5	159 (21%)
	6	24 (3%)
	missing	2
CTS-6 (1 to 5)	Mean (SD)	2.89 (0.85)
MHQ overall function (0-100)	Mean (SD)	53.4 (22.6)
MHQ unilateral activities (0-100)	Mean (SD)	66.6 (28.6)
MHQ bilateral activities (0-100)	Mean (SD)	69.4 (25.5)
MHQ work (0-100)	Mean (SD)	64.7 (25.6)
HADS Anxiety	Mean (SD)	6.18 (4.52)
	Normal (1-7)	497 (66%)
	Mild (8-11)	123 (16%)
	Moderate (11-14)	90 (12%)
	Severe (15-21)	42 (6%)
	Missing	1
HADS Depression	Mean (SD)	4.48 (3.84)
	Normal (1-7)	602 (80%)
	Mild (8-10)	88 (12%)
	Moderate (11-14)	47 (6%)
	Severe (15-21)	15 (2%)
	Missing	1
Overall Co-morbidity Score (0-36)	Mean (SD)	5.22 (4.17)
EQ5D-3L VAS	Mean (SD)	73.54 (18.2)

For peer review only

For BMJ Open

Table 3: Self-reported Comorbidities

Comorbidity	Has problem	Receives treatment	Limits activity
Back pain	342 (45%)	142 (19%)	226 (30%)
Osteoarthritis	253 (34%)	129 (17%)	185 (25%)
High Blood Pressure	235 (31%)	226 (30%)	22 (3%)
Depression	148 (20%)	117 (16%)	63 (8%)
Diabetes	77 (10%)	63 (8%)	11 (1%)
Rheumatoid Arthritis	70 (9%)	40 (5%)	51 (7%)
Lung disease	29 (4%)	23 (3%)	18 (2%)
Cancer	18 (2%)	13 (2%)	5 (<1%)
Kidney disease	14 (2%)	5 (<1%)	3 (<1%)
Liver disease	4 (<1%)	1 (<1%)	0
Ulcer or stomach disease	37 (5%)	32 (4%)	7 (1%)
Other Condition 1	372 (49%)	280 (37%)	150 (20%)
Other Condition 2	140 (19%)	95 (13%)	60 (8%)
Other Condition 3	48 (6%)	30 (4%)	20 (3%)

For peer review only

	n	HADS ANX Mean (SD)	Estimate (95% CI)[1]	HADS DEP Mean (SD)	Estimate (95% CI)[1]	EQ-5D Mean (SD)	Estimate (95% CI)[1]
CTS 6 Score							
1.00	7	3.14 (2.61)		1.57 (1.81)		0.78 (0.32)	
1.01 – 2.00	134	5.04 (4.07)		2.90 (3.12)		0.77 (0.20)	
2.01 – 3.00	308	5.50 (4.05)		3.86 (3.31)		0.72 (0.20)	
3.01 – 4.00	241	6.87 (4.55)		5.42 (3.99)		0.60 (0.28)	
4.01 – 5.00	63	9.62 (5.48)	1.11 (0.73, 1.49) P<0.0001 R^2 = 17.7%	7.68 (4.44)	1.43 (1.13, 1.73) P<0.0001 R^2 = 26.1%	0.43 (0.34)	-0.10 (-0.12, -0.08) P<0.0001 R^2 = 37.4%
NCS Grade							
1	150	6.78 (4.79)		4.64 (4.10)		0.66 (0.28)	
2	79	7.13 (4.78)		4.78 (3.73)		0.68 (0.25)	
3	202	6.44 (4.36)		4.33 (3.77)		0.68 (0.27)	
4	137	6.24 (4.54)		4.80 (3.92)		0.68 (0.26)	
5	159	5.18 (4.24)		4.20 (3.80)		0.66 (0.24)	
6	24	3.42 (3.09)	-0.26 (-0.49, -0.03) P=0.029 R^2 = 14.3%	4.08 (3.13)	-0.00 (-0.20, 0.20) P=0.983 R^2 = 17.1%	0.61 (0.26)	-0.01 (-0.03, 0.01) P=0.214 R^2 = 28.4%

Table 4(a) Relationship between CTS Severity and Anxiety, Depression and Quality of Life

1: Parameter estimate (95% confidence interval) from a linear model including gender, age, ethnicity, employment status, smoking status, units of alcohol per week, BMI, duration of CTS and comorbidity score.

For peer review only

For BMJ Open

Table 4(b) Relationship between CTS Severity and MHQ sub-scales

	n	Overall Function Mean (SD)	Estimate (95% CI)[1]	ADL unilateral Mean (SD)	Estimate (95% CI)[1]	ADL bilateral Mean (SD)	Estimate (95% CI)[1]	Work Mean (SD)	Estimate (95% CI)[1]
CTS 6 Score									
1.00	7	92.1 (10.7)		91.4 (16.5)		88.8 (11.4)		92.9 (12.9)	
1.01 – 2.00	134	68.5 (20.3)		82.9 (22.0)		82.9 (18.9)		78.7 (22.3)	
2.01 – 3.00	308	58.3 (19.1)		74.5 (23.1)		76.2 (20.7)		70.7 (22.8)	
3.01 – 4.00	241	44.9 (18.9)		56.8 (26.3)		60.7 (24.9)		55.0 (23.2)	
4.01 – 5.00	63	25.8 (16.2)	-14.7 (-16.4, -13.1) P<0.0001 R^2 = 41.1%	28.2 (26.4)	-17.8 (-19.9, -15.8) P<0.0001 R^2 = 40.7%	38.3 (26.2)	-14.6 (-16.4, -12.8) P<0.0001 R^2 = 40.8%	39.6 (22.2)	-13.4 (-15.4, -11.5) P<0.0001 R^2 = 33.3%
NCS Grade									
1	150	58.9 (23.9)		71.0 (27.9)		72.7 (24.4)		63.9 (27.4)	
2	79	53.5 (22.9)		71.5 (27.7)		74.1 (25.2)		67.5 (26.2)	
3	202	54.6 (22.8)		70.5 (27.7)		72.6 (24.6)		65.8 (26.0)	
4	137	53.1 (20.5)		67.0 (26.3)		68.2 (24.3)		63.4 (23.9)	
5	159	48.8 (21.2)		57.8 (30.0)		63.5 (27.3)		64.4 (24.4)	
6	24	43.3 (24.9)	-3.16 (-3.56, -1.22) P<0.0001 R^2 = 15.5%	47.9 (29.8)	-3.87 (-5.32, -2.42) P<0.0001 R^2 = 18.7%	52.5 (26.0)	-3.16 (-4.42, -1.90) P<0.0001 R^2 = 21.9%	61.9 (23.6)	-0.86 (-2.18,0.47) P=0.197 R^2 = 15.7%

[1] Parameter estimate (95% confidence interval) from a linear model including gender, age, ethnicity, employment status, smoking status, units of alcohol per week, BMI, duration of CTS and comorbidity score. ADL: activities of daily living

For peer review only - http://bmjopen.bmj.com/site/about/guidelines.xhtml

For BMJ Open

	n	NHS cost (SD)	exp(b)(95% CI)[1]	Societal cost (SD)	exp(b)(95% CI)[1]
CTS-6					
1.00	7	£512.83 (£405.61)		£726.02 (£776.80)	
1.01-2.00	134	£434.04 (£212.53)		£559.37 (£430.53)	
2.01-3.00	308	£422.57 (£220.30)		£554.73 (£492.25)	
3.01-4.00	241	£466.50 (£348.74)		£686.11 (£814.25)	
4.01-5.00	63	£511.52 (£273.65)	1.08 (1.03, 1.12)	£984.19 (£1,197.94)	1.18 (1.10, 1.27)

For peer review only

Table 5: Relationship between CTS Severity, Age and Cost

For BMJ Open

			**p<0.01**		**p<0.0001**
NCS Grade					
1	150	£483.36 (£456.12)		£629.69 (£608.90)	
2	79	£459.44 (£243.93)		£592.70 (£381.55)	
3	202	£438.50 (£213.31)		£657.58 (£755.06)	
4	137	£414.14 (£153.80)		£578.23 (£597.95)	
5	159	£439.38 (£203.02)		£676.24 (£892.99)	
6	24	£467.71 (£237.77)	0.98 (0.96, 1.01) p=0.269	£583.77 (£294.98)	1.01 (0.96, 1.07) P=0.590
Age & CTS-6					
Age 18 – 40	43	£433.41 (£178.60)		£758.67 (£1,099.72)	
Age 41 – 60	344	£444.53 (£226.94)	1.04 (0.88, 1.22) p=0.679	£613.03 (£533.45)	0.83 (0.63, 1.10) p=0.196
Age 61 – 80	322	£451.96 (£336.62)	1.13 (0.94, 1.36) p=0.190	£631.64 (£731.18)	0.90 (0.66, 1.22) p=0.486
Age 81 plus	44	£442.46 (£146.20)	1.16 (0.91, 1.47) p=0.224	£712.66 (£973.69)	1.09 (0.73, 1.62) p=0.690
Age & NCS Grade					
Age 18 – 40	43	£433.41 (£178.60)		£758.67 (£1,099.72)	
Age 41 – 60	343	£443.81 (£226.88)	1.02 (0.87, 1.21) p=0.774	£608.42 (£527.83)	0.81 (0.60, 1.09) p=0.163
Age 61 – 80	321	£451.29 (£336.93)	1.11 (0.93, 1.34) p=0.246	£629.87 (£730.63)	0.85 (0.61, 1.18) p=0.336
Age 81 plus	44	£442.46 (£146.20)	1.15 (0.90, 1.47) p=0.252	£712.65 (£973.69)	0.99 (0.64, 1.53) p=0.976

[1] Exponentiated parameter estimate (standard error) from a GLM model with log-link and gamma distributed errors including gender, age, ethnicity, employment status, smoking status, units of alcohol per week, BMI, bilateral CTS, co-morbidities and comorbidity score.

For peer review only

For BMJ Open

References

1. Padua L, Coraci D, Erra C, et al. Carpal tunnel syndrome: clinical features, diagnosis, and management. The Lancet Neurology;**15**(12):1273-84.
2. Bland JDP. Carpal tunnel syndrome. Curr Opin Neurol 2005;**18**(5):581-85.
3. Latinovic R, Gulliford MC, Hughes RAC. Incidence of common compressive neuropathies in primary care. J Neurol Neurosur Psych 2006;**77**(2):263-65.
4. Meys V, Thissen S, Rozeman S, et al. Prognostic factors in carpal tunnel syndrome treated with a corticosteroid injection. Muscle & Nerve 2011;**44**(5):763-8.
5. Hameso A, Bland JD. Prevalence of decompression surgery in patients with carpal tunnel syndrome 8 years after initial treatment with a local corticosteroid injection. J Hand Surg, European Volume 2017;**42**(3):275-80.
6. Bebbington E, Furniss D. Linear regression analysis of Hospital Episode Statistics predicts a large increase in demand for elective hand surgery in England. J Plast Reconstr Aes 2015;**68**(2):243-51.
7. Ryan D, Shaw A, Graham S, et al. Variation in CCG policies for the treatment of carpal tunnel syndrome. RCS Bulletin 2017;**99**(1):28-31.
8. Duckworth AD, Jenkins PJ, Roddam P, et al. Pain and carpal tunnel syndrome. The Journal of Hand Surg 2013;**38**(8):1540-6.
9. Nunez F, Vranceanu AM, Ring D. Determinants of pain in patients with carpal tunnel syndrome. Clin OrthRel Res 2010;**468**(12):3328-32.
10. Fernandez-de-las-Penas C, Fernandez-Munoz JJ, Palacios-Cena M, et al. Direct and Indirect Effects of Function in Associated Variables Such as Depression and Severity on Pain Intensity in Women with Carpal Tunnel Syndrome. Pain Medicine 2015;**16**(12):2405-11.
11. Jerosch-Herold C, Shepstone L, Wilson EC, et al. Clinical course, costs and predictive factors for response to treatment in carpal tunnel syndrome: the PALMS study protocol. BMC Musc Dis 2014;**15**:35.
12. Bland JDP. A neurophysiological grading scale for carpal tunnel syndrome. Muscle & Nerve 2000;**23**(8):1280-83.
13. Levine D, Simmons B, Koris M, et al. A self-administered questionnaire for the assessment of severity of symptoms and functional status in carpal tunnel syndrome. J Bone Joint Surg Am 1993;**75A**(11):1585-92.
14. Atroshi I, Lyren PE, Gummesson C. The 6-item CTS symptoms scale: a brief outcomes measure for carpal tunnel syndrome. Qual Life Res 2009;**18**(3):347-58.
15. Chung KC, Pillsbury MS, Walters MR, et al. Reliability and validity testing of the Michigan Hand Outcomes Questionnaire. J Hand Surg-Am 1998;**23A**(4):575-87.
16. Kotsis SV, Chung KC. Responsiveness of the Michigan Hand Outcomes Questionnaire and the Disabilities of the Arm, Shoulder and Hand Questionnaire in carpal tunnel surgery. J Hand Surg-Am 2005;**30A**(1):81-86.
17. Snaith RP, Zigmond AS. The Hospital Anxiety and Depression Scale. Brit Med J 1986;**292**(6516):344-44.
18. EuroQol Group. EuroQol-a new facility for the measurement of health-related quality of life. Health Policy 1990;**16**(3):199-208.
19. Sangha O, Stucki G, Liang MH, et al. The self-administered comorbidity questionnaire: A new method to assess comorbidity for clinical and health services research. Arthrit Rheum-Arthr 2003;**49**(2):156-63.
20. Dolan P. Modeling valuations for EuroQol health states. Med Care 1997;**35**(11):1095-108.
21. Hobby JL, Venkatesh R, Motkur P. The effect of psychological disturbance on symptoms, self-reported disability and surgical outcome in carpal tunnel syndrome. J Bone Joint Surg Br 2005;**87B**(2):196-200.

For peer review only - http://bmjopen.bmj.com/site/about/guidelines.xhtml

For BMJ Open

22. Sears ED, Swiatek PR, Hou H, et al. Utilization of Preoperative Electrodiagnostic Studies for Carpal Tunnel Syndrome: An Analysis of National Practice Patterns. J Hand Surg-Am 2016;**41**(6):665-72.
23. Thomson JG. Diagnosis and treatment of carpal tunnel syndrome - correspondence. Lancet Neurol;**16**(4):263.
24. Padua L, Coraci D, Erra C, et al. Diagnosis and treatment of carpal tunnel syndrome - Authors' response. Lancet Neurol;**16**(4):263-64.
25. Chan L, Turner JA, Comstock BA, et al. The relationship between electrodiagnostic findings and patient symptoms and function in carpal tunnel syndrome. Arch Phys Med Rehab 2007;**88**(1):19-24.
26. Schmid AB, Bland JDP, Bhat MA, et al. The relationship of nerve fibre pathology to sensory function in entrapment neuropathy. Brain 2014;**137**:3186-99.
27. Povlsen B, Aggelakis K, Koutroumanidis M. Effect of age on subjective complaints and objective severity of carpal tunnel syndrome: prospective study. JRSM Short Reports 2010;**1**(7):62.
28. Hobby JL, Venkatesh R, Motkur P. The effect of age and gender upon symptoms and surgical outcomes in carpal tunnel syndrome. J Hand Surg-Brit Eur 2005;**30B**(6):599-604.
29. Bland JDP. Do nerve conduction studies predict the outcome of carpal tunnel decompression? Muscle & Nerve 2001;**24**(7):935-40.
30. Nazari G, MacDermid JC, Bain J, et al. Estimation of health-related-quality of life depends on which utility measure is selected for patients with carpal tunnel syndrome. J Hand Ther published online December 14, 2016:1-7.
31. Marti C, Hensler S, Herren DB, et al. Measurement properties of the EuroQoL EQ-5D-5L to assess quality of life in patients undergoing carpal tunnel release. J Hand Surg, Europ Vol 2016; **41**(9):957-962.
32. Atroshi I, Gummesson C, McCabe SJ, et al. The SF-6D health utility index in carpal tunnel syndrome. J Hand Surg, Europ Vol 2007;**32**(2):198-202.
33. Dyer MT, Goldsmith KA, Sharples LS, et al. A review of health utilities using the EQ-5D in studies of cardiovascular disease. Health Qual of Life Outcomes 2010;**8**(1):13.
34. Hakkaart L, Lamers LM, Bouwmans CAM, et al. Comparison of EQ-5D and SF-6D in mental health patients. J Ment Health Policy 2007;**10**:S17-S17.
35. Pickard AS, Wilke CT, Lin HW, et al. Health utilities using the EQ-5D in studies of cancer. Pharmacoeconomics 2007;**25**(5):365-84.

Figure Legends

Figure 1 : STROBE flowchart

For peer review only

For peer review only - http://bmjopen.bmj.com/site/about/guidelines.xhtml

Figure 1 : STROBE flowchart [31]

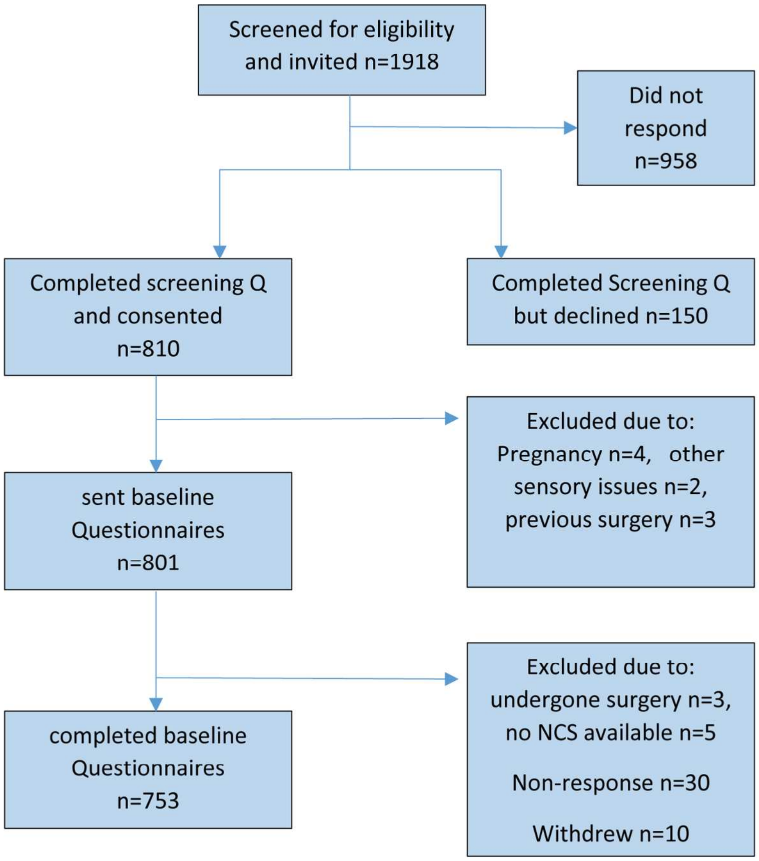

Figure1: STROBE flowchart

156x142mm (300 x 300 DPI)

Supplementary File I:

Criteria for grading of nerve conduction studies:

Grade 0	Normal	No neurophysiological abnormality
Grade 1	Very mild	Median SAP digit II or III to wrist: < 50m/s and ≥40 m/s, Ulnar SAP>50m/s<br>Median DML APB to wrist: >4.0ms and Ulnar DML <=3.8ms<br>or<br>2 sensitive tests positive (ring finger 'double peak' SAP or palm/wrist – median/ulnar comparison or Lumbrical to interosseous latency increase)
Grade 2	Mild	Median SAP digit II or III to wrist SAP: < 40 m/sec<br>Median DML APB to wrist: < 4.5msec
Grade 3	Moderate	Median SAP digit II or III to wrist: present<br>Median DML APB to wrist: > 4.5msec and < 6.5 ms
Grade 4	Severe	Median SAP digit II or III to wrist: absent<br>Median DML APB to wrist: > 4.5 and < 6.5 ms
Grade 5	Very severe	Median DML APB to wrist : > 6.5 msec
Grade 6	Extremely severe	Median DML APB to wrist: unrecordable (< 0.2 mV peak to peak)

SAP: sensory action potential, DML: distal motor latency; APB: Abductor Pollicis Brevis; ms: milliseconds

For peer review only

For peer review only

STROBE Statement—checklist of items that should be included in reports of observational studies

	Item No	Recommendation	Page
**Title and abstract**	1	(*a*) Indicate the study's design with a commonly used term in the title or the abstract	**3**
		(*b*) Provide in the abstract an informative and balanced summary of what was done and what was found	**3**
**Introduction**			
Background/rationale	2	Explain the scientific background and rationale for the investigation being reported	**4**
Objectives	3	State specific objectives, including any prespecified hypotheses	**4**
**Methods**			
Study design	4	Present key elements of study design early in the paper	**3/4**
Setting	5	Describe the setting, locations, and relevant dates, including periods of recruitment, exposure, follow-up, and data collection	**4/5**
Participants	6	(*a*) *Cohort study*—Give the eligibility criteria, and the sources and methods of selection of participants. Describe methods of follow-up<br>*Case-control study*—Give the eligibility criteria, and the sources and methods of case ascertainment and control selection. Give the rationale for the choice of cases and controls<br>*Cross-sectional study*—Give the eligibility criteria, and the sources and methods of selection of participants	**5**
		(*b*) *Cohort study*—For matched studies, give matching criteria and number of exposed and unexposed<br>*Case-control study*—For matched studies, give matching criteria and the number of controls per case	
Variables	7	Clearly define all outcomes, exposures, predictors, potential confounders, and effect modifiers. Give diagnostic criteria, if applicable	**5/6**
Data sources/ measurement	8*	For each variable of interest, give sources of data and details of methods of assessment (measurement). Describe comparability of assessment methods if there is more than one group	**6**
Bias	9	Describe any efforts to address potential sources of bias	**6**
Study size	10	Explain how the study size was arrived at	**n/a**
Quantitative variables	11	Explain how quantitative variables were handled in the analyses. If applicable, describe which groupings were chosen and why	**6**
Statistical methods	12	(*a*) Describe all statistical methods, including those used to control for confounding	**6**
		(*b*) Describe any methods used to examine subgroups and interactions	**6**
		(*c*) Explain how missing data were addressed	**6**
		(*d*) *Cohort study*—If applicable, explain how loss to follow-up was addressed<br>*Case-control study*—If applicable, explain how matching of cases and controls was addressed<br>*Cross-sectional study*—If applicable, describe analytical methods taking account of sampling strategy	**5/6**
		(*e*) Describe any sensitivity analyses	

For peer review only - http://bmjopen.bmj.com/site/about/guidelines.xhtml

Results			page
Participants	13*	(a) Report numbers of individuals at each stage of study—eg numbers potentially eligible, examined for eligibility, confirmed eligible, included in the study, completing follow-up, and analysed	**6**
		(b) Give reasons for non-participation at each stage	
		(c) Consider use of a flow diagram	**Fig 1**
Descriptive data	14*	(a) Give characteristics of study participants (eg demographic, clinical, social) and information on exposures and potential confounders	**12**
		(b) Indicate number of participants with missing data for each variable of interest	**12**
		(c) *Cohort study*—Summarise follow-up time (eg, average and total amount)	**n/a**
Outcome data	15*	*Cohort study*—Report numbers of outcome events or summary measures over time	
		Case-control study—Report numbers in each exposure category, or summary measures of exposure	
		Cross-sectional study—Report numbers of outcome events or summary measures	**12/13**
Main results	16	(*a*) Give unadjusted estimates and, if applicable, confounder-adjusted estimates and their precision (eg, 95% confidence interval). Make clear which confounders were adjusted for and why they were included	**7, 15, 16**
		(*b*) Report category boundaries when continuous variables were categorized	**n/a**
		(*c*) If relevant, consider translating estimates of relative risk into absolute risk for a meaningful time period	**n/a**
Other analyses	17	Report other analyses done—eg analyses of subgroups and interactions, and sensitivity analyses	
**Discussion**			
Key results	18	Summarise key results with reference to study objectives	**8**
Limitations	19	Discuss limitations of the study, taking into account sources of potential bias or imprecision. Discuss both direction and magnitude of any potential bias	**10**
Interpretation	20	Give a cautious overall interpretation of results considering objectives, limitations, multiplicity of analyses, results from similar studies, and other relevant evidence	**10**
Generalisability	21	Discuss the generalisability (external validity) of the study results	**10**
**Other information**			
Funding	22	Give the source of funding and the role of the funders for the present study and, if applicable, for the original study on which the present article is based	**1**

*Give information separately for cases and controls in case-control studies and, if applicable, for exposed and unexposed groups in cohort and cross-sectional studies.

Note: An Explanation and Elaboration article discusses each checklist item and gives methodological background and published examples of transparent reporting. The STROBE checklist is best used in conjunction with this article (freely available on the Web sites of PLoS Medicine at http://www.plosmedicine.org/, Annals of Internal Medicine at http://www.annals.org/, and Epidemiology at http://www.epidem.com/). Information on the STROBE Initiative is available at www.strobe-statement.org.

For peer review only

For peer review only - http://bmjopen.bmj.com/site/about/guidelines.xhtml

BMJ Open

Association of psychological distress, quality of life and costs with carpal tunnel syndrome severity: a cross-sectional analysis of the PALMS cohort

Journal:	*BMJ Open*
Manuscript ID	bmjopen-2017-017732.R3
Article Type:	Research
Date Submitted by the Author:	11-Sep-2017
Complete List of Authors:	Jerosch-Herold, Christina; University of East Anglia, Health Sciences<br>Houghton, Julie; University of East Anglia Faculty of Medicine and Health Sciences, Health Sciences<br>Blake, Julian; Norfolk and Norwich University Hospitals NHS Foundation Trust, Neurophysiology<br>Shaikh, Anum; University of Cambridge Department of Public Health and Primary Care<br>Wilson, Edward; University of Cambridge, Cambridge Centre for Health Services Research<br>Shepstone, Lee; University of East Anglia Faculty of Medicine and Health Sciences
**Primary Subject Heading**:	Health services research
Secondary Subject Heading:	Health economics, Mental health, General practice / Family practice, Neurology
Keywords:	Neurological pain < NEUROLOGY, Neurophysiology < NEUROLOGY, Hand & wrist < ORTHOPAEDIC & TRAUMA SURGERY, Musculoskeletal disorders < ORTHOPAEDIC & TRAUMA SURGERY, PRIMARY CARE, Depression & mood disorders < PSYCHIATRY

SCHOLARONE™
Manuscripts

For peer review only - http://bmjopen.bmj.com/site/about/guidelines.xhtml

For BMJ Open

Title

Association of psychological distress, quality of life and costs with carpal tunnel syndrome severity: a cross-sectional analysis of the PALMS cohort

Authors

Christina Jerosch-Herold[1]

Julie Houghton[1]

Julian Blake[2, 5]

Anum Shaikh[3]

Edward CF Wilson[3]

Lee Shepstone[4]

Affiliations:

[1]School of Health Sciences, Faculty of Medicine and Health Sciences, University of East Anglia, Norwich, Norfolk, United Kingdom.

[2]Department of Clinical Neurophysiology, Norfolk and Norwich University Hospitals NHS foundation Trust, Norwich, UK

[3] Cambridge Centre for Health Services Research, Institute of Public Health, University of Cambridge, Cambridge, UK

[4]Norwich Medical School, Faculty of Medicine and Health Sciences, University of East Anglia, Norwich, Norfolk, United Kingdom.

[5]MRC Centre for Neuromuscular Diseases, Queen Square, London,

Corresponding author:

Dr Christina Jerosch-Herold, School of Health Sciences, Faculty of Medicine and Health Sciences, University of East Anglia, Norwich, Norfolk, NR4 7TJ United Kingdom. Email: c.jerosch-herold@uea.ac.uk

Funding:

CJH was funded by the National Institute for Health Research (NIHR) through a NIHR Senior Research Fellowship. ECFW is funded by the NIHR Cambridge Biomedical Research Centre. The funders of the study had no role in study design, data collection, data analysis, data interpretation, or writing of the report. The views and opinions expressed therein are those of the authors and do not necessarily reflect those of the NIHR, NHS or the Department of Health.

Conflict of interest:

We affirm that we have no financial affiliation (including research funding) or involvement with any commercial organization that has a direct financial interest in any matter included in this manuscript, except as disclosed in an attachment and cited in the manuscript.

For peer review only - http://bmjopen.bmj.com/site/about/guidelines.xhtml

For peer review only

For BMJ Open

Acknowledgements:

We thank the patients who participated in this study; the local Principal Investigators at the following collaborating NHS sites: Julian Blake and Adrian Tearle (Norfolk and Norwich University Hospitals NHS Trust), Ann Harvey (Ipswich Hospital), Stephen Scott (James Paget Hospital, Gt Yarmouth), Jeremy Bland (Kings College Hospital), Lionel Christopher Bainbridge (Royal Derby Hospital); lay members from the local Patient and Public Involvement in Research group: Jenny Griffiths and Sue Spooner; Tony Dyer and Antony Colles for database support (Norwich Clinical Trials Unit)

Contributorship:

CJH conceived the PALMS cohort study and obtained funding. CJH, JB, LS and ECFW developed the protocol. JH conducted data collection and data entry. LS, AS and ECFW undertook the statistical analyses. All authors contributed to the drafting of the manuscript and have read and approved the final manuscript.

Data Sharing Statement: no additional data are available

For peer review only

For BMJ Open

Abstract

Objectives:

The PALMS study is designed to identify prognostic factors for outcome from corticosteroid injection and surgical decompression for carpal tunnel syndrome (CTS) and predictors of cost over 2 years. The aim of this paper is to explore the cross-sectional association of baseline patient-reported and clinical severity with anxiety, depression, health-related quality of life and costs of CTS in patients referred to secondary care.

Methods:

Prospective, multi-centre cohort study initiated in 2013. We collected baseline data on patient-reported symptom severity (CTS-6), psychological status (HADS), hand function (Michigan Hand Questionnaire) comorbidities, EQ5D-3L and sociodemographic variables. Nerve conduction tests classified patients into five severity grades (mild to very severe). Data were analysed using a general linear model.

Results:

753 patients with CTS provided complete baseline data. Multivariable linear regression adjusting for age, sex, ethnicity, duration of CTS, smoking status, alcohol consumption, employment status, body mass index and comorbidities showed a highly statistically significant relationship between CTS-6 and anxiety, depression and the EQ-5D ($p<0.0001$ in each case). Likewise, a significant relationship was observed between electrodiagnostic severity and anxiety ($p=0.027$) but not with depression ($p=0.986$) or the EQ-5D ($p=0.257$). NHS and societal costs in the 3 months prior to enrolment were significantly associated with self-reported severity ($p<0.0001$) but not with electrodiagnostic severity.

Conclusions:

Patient-reported symptom severity in carpal tunnel syndrome is significantly and positively associated with anxiety, depression, health-related quality of life and NHS and societal costs even when adjusting for age, gender, body mass index, comorbidities, smoking, drinking and occupational status. In contrast there is little or no evidence of any relationship with objectively derived CTS severity. Future research is needed to understand the impact of approaches and treatments that address psychosocial stressors as well as biomedical factors on relief of symptoms from carpal tunnel syndrome.

Article Summary – Strengths and limitations

- Large multi-centre prospective cohort study of CTS (n>700)
- We used a multivariable model to test the independence of associations with a range of pre-specified demographic and clinical variables
- All patients had a diagnosis of CTS made through the combination of clinical history, signs and symptoms with objective electrodiagnostic testing
- Cross-sectional analysis limited to baseline data only with longitudinal (2 year) follow-up still in progress.
- Cost data is limited to a 3 months horizon prior to enrolment

For peer review only - http://bmjopen.bmj.com/site/about/guidelines.xhtml

For peer review only

For BMJ Open

Introduction

Carpal tunnel syndrome (CTS) is the most common nerve entrapment syndrome; an estimated 1 in 10 people are likely to develop symptoms at some stage in their lives [1]. CTS is characterised by pins and needles, pain and numbness, often affecting both hands. Symptoms can range from intermittent to constant and from very mild to very severe, potentially interfering significantly with daily activities[2]. Approximately 1/3rd to 2/3rd of patients with CTS go on to have surgery [3 4 5]. Such variations in surgical rates may be due to different follow-up periods in individual studies as well as differences in criteria for and access to surgical decompression.

54,000 carpal tunnel decompressions were carried out in England in 2014-15 (Hospital Episode Statistics) at a cost of £46 million to the NHS (based on an NHS tariff of £864) and are predicted to increase two-fold by 2030 [6]. Clinical Commissioning Groups (CCGs) in England have introduced guidelines on funding for procedures deemed to be of 'limited clinical value', recommending carpal tunnel surgery only for those with '*moderate to severe CTS*' or who have '*not responded to conservative measures*' (ie corticosteroid injections and splints). However it is often unclear whether severity refers to pathophysiology or symptoms or a combination of both. A recent review of policies from 175 CCGs in England highlighted that these were highly variable in terms of criteria for defining severity, the necessity for nerve conduction studies (NCS) and duration of non-operative treatment before referral for surgery is allowed [7]. Controversy continues over the best management of CTS at different stages. More recent research on CTS has highlighted the role of psychological factors such as anxiety, depression and patient's health beliefs in how patients perceive their symptoms, their impact on function and the outcome from treatment [8-10]. No previous studies have explored the association of disease and symptom severity with health-related quality of life or costs from a personal and societal perspective and which may also play an important role in treatment planning.

The objectives of this cross-sectional analysis were i) to explore the association of self-reported symptom severity and electrodiagnostic severity with anxiety, depression, hand function and health-related quality of life in patients referred for diagnosis and treatment of CTS; and ii) to describe NHS and societal costs of CTS in the 3 months preceding referral for nerve conduction testing and treatment and their association with carpal tunnel severity.

Methods

For peer review only - http://bmjopen.bmj.com/site/about/guidelines.xhtml

For BMJ Open

Design and study population

A prospective longitudinal cohort study (PALMS study) was initiated in 2013 to develop a multivariable prognostic model of predictive factors for outcome after steroid injection, outcome after surgery and costs in CTS. The full study protocol has been described elsewhere [11]. Recruitment took place at five secondary care sites across five NHS Trusts in England where patients had been referred by primary care for nerve conduction tests and prior to any treatment. Patients were diagnosed with CTS by a neurophysiologist or hand surgeon based on signs and symptoms, clinical history and objective measurable pathophysiology (NCS). Eligible patients were invited to participate by returning completed screening questionnaires and signed consent forms. Patients were included if they had CTS in at least one hand confirmed by NCS (grade ≥1) and were aged 18 years or over. NCS grading was done according Bland's criteria[12] (for details see Supplementary Table I). Patients with concomitant diseases such as diabetes or hypothyroidism were included. Exclusion criteria were previous surgery in the affected hand in the last 12 months, pregnancy or up to 12-months post-partum, serious comorbidities, other limb mononeuropathies, sensory or motor disturbances secondary to stroke, multiple sclerosis or nerve injury and inability to speak or write English.

For the present study data collected at baseline were used. Self-reported symptom severity and NCS for only one hand per patient was used. In patients with bilateral CTS the 'index hand' was defined as either the worst hand or the dominant hand where self-reported symptom severity on the CTS-6 was the same bilaterally.

Ethical approval was given by the NRES Committee East of England – Norfolk on 22nd April 2013 (reference 13/EE/0106) and local Research Governance approval at each participating trust was obtained prior to recruitment.

Data collection

A baseline questionnaire was developed comprising the following standardised and non-standardised questionnaires:

- Symptom severity was assessed with the 6-item shortened form of the Boston Carpal Tunnel Questionnaire (BCTQ) [13], the CTS-6 [14] for each hand. The scores range from 1 (no symptoms) to 5 (very severe symptoms). A mean score of 1 indicates no symptoms (tingling, numbness and pain during the past 2 weeks).
- Hand functional status was assessed with 3 sub-scales of the Michigan Hand Questionnaire (MHQ) [15,16] : overall hand function for worst hand, unilateral and bilateral activities of daily

For peer review only - http://bmjopen.bmj.com/site/about/guidelines.xhtml

living and work performance. Each subscale is converted into a percentage with higher values denoting greater disability.

- Psychological status was assessed using the Hospital Anxiety and Depression Scale (HADS) [17]. It is made up of 14 items, 7 relating to anxiety and 7 relating to depression. Responses are scored from 0-3 giving a possible score range of 0 to 21 for each subscale. Scores of 8 to 10 identify mild cases, 11 to 15 moderate cases and 16 or more, severe cases. [17]
- Health-related quality of life (HRQoL) was assessed by the EuroQoL EQ5D-3L[18].
- Comorbidities were collected by the Self-Administered Comorbidity Questionnaire (SACQ) [19]. The total score ranges from 0 to 36 which is based on 12 listed conditions for which the patient indicates if they have it (1 point), receive treatment for it (2 points) and whether it is activity limiting (3 points).
- Health care resource use in the 3 months prior to enrolment. This included questions about treatments received (including alternative therapies), medications (prescription and over-the-counter), healthcare contacts (NHS primary, secondary and tertiary, and private), patient-reported days off work due to CTS and assistance with activities of daily living due to CTS.

Other clinical and demographic variables collected at baseline were age, sex, duration of symptoms, height and weight, work status and type, smoking status, alcohol units consumed per week, ethnicity and household income. Additionally, the full NCS reports of enrolled patients were obtained from the participating sites and were graded by the first author using Bland's criteria[12] from 1 (mild) to 6 (extremely severe) (see Supplementary Table I)

Patients were given the option of completing the study questionnaires online via a personalised link to a password protected web-based data entry system maintained by the Norwich Clinical Trials Unit or via paper-based questionnaires sent by mail.

Statistical analysis

A general linear model (GLM) with a normally distributed error term was used to model the HADS anxiety score, HADS depression score, MHQ scores and EQ-5D utility index. EQ-5D responses were converted to utilities using UK-specific preference weights [20]. The model included gender, age, ethnicity, employment status, smoking status, units of alcohol per week, body mass index (BMI), duration of CTS and comorbidity score as explanatory variables before adding either CTS-6 symptom

For peer review only - http://bmjopen.bmj.com/site/about/guidelines.xhtml

For peer review only

score or NCS score. Parameter estimates for the effect of CTS-6 and NCS score are provided with 95% confidence intervals and statistical significance set at the conventional 5% level.
Resource use data were converted to cost per patient using standard UK unit cost sources [Personal Social Services Research Unit (PSSRU), British National Formulary (BNF), and Office for National Statistics (ONS)], the relevant published literature and consultation with experts. Costs were then modelled using a GLM with log link and gamma distributed errors. The base year for the costs was 2015/2016 and the analysis was conducted from the perspectives of the NHS and society (defined as the sum of NHS and patient out-of-pocket costs and morbidity-related lost productivity).

Results

Recruitment took place over 30 months between July 2013 and December 2015. A total of 1918 patients with CTS were identified and invited to participate of which a total of 753 patients met all eligibility criteria and returned full baseline questionnaires (see Strobe diagram in Figure 1).

The sociodemographic and clinical variables of the cohort are summarised in Tables 1 and 2. Bilateral CTS affected 69% of patients and 75% of participants had their symptoms for at least 6 months or longer. Using their worst hand as the index hand only 18% had received a steroid injection and mean self-reported symptom severity (CTS-6) was 2.89 points. Based upon nerve conduction tests, 69% of patients had at least moderate CTS (grades ≥3), with 24% in the severe and very severe categories (grades 5 or 6) (see Table 2).

The mean CTS-6 scores increased steadily with NCS grade, up to grade 6 (which contained relatively few individuals). The mean (and standard deviation) by grade were as follows: grade 1, 2.69 (0.86); grade 2, 2.79 (0.85); grade 3, 2.87 (0.91); grade 4, 3.00 (0.79); grade 5, 3.06 (0.79); grade 6, 2.89 (0.78).

The HADS anxiety and HADS depression scores were within normal ranges (score 0 to 7) in 66% and 80% of participants, respectively. Self-reported comorbidities are summarised in Table 3. The mean SACQ score was 5.22. The three commonest co-morbidities were low back pain (LBP), osteoarthritis (OA) and high blood pressure with LBP and OA also reported as the most activity limiting. Mean VAS score on the EQ5D-3L was 73.5 (SD= 18.2), and the mean health utility index was 0.66. Over 85% of respondents indicated having at least some pain and 31% of the cohort indicated having both pain and feeling anxious or depressed.

Using a multivariable regression model including age, sex, ethnicity, duration of CTS, smoking status, alcohol consumption, employment status, BMI and comorbidity score as potential confounding

For peer review only - http://bmjopen.bmj.com/site/about/guidelines.xhtml

variables, a highly statistically significant association was found between self-reported symptom severity (using CTS-6 score) and anxiety, depression and HRQoL ($p<0.0001$ in each case). Table 4a gives the parameter estimates for psychological outcome and quality of life by self-reported and objectively graded severity. For each 1 point increase in score on the CTS-6 there was an estimated 1.11 points increase in anxiety score, 1.43 points increase in depression score and a 10% mean decrease in health utility. Patients with the highest symptom severity (4 to 5 points on CTS-6) had mean anxiety scores of 9.62 (SD=5.48) indicating at least mild anxiety. There was a marked lower health utility for those with the highest symptom severity (score 4 to 5 on CTS-6) with a mean health utility index of 0.43 compared to 0.77 in the mildest group (score 1 to 2 on CTS-6). When including the same independent variables in the model, objective severity grading based on NCS was significantly associated with anxiety ($p=0.027$), but not with depression or HRQoL. The association between NCS grade and anxiety, however, was negative with higher anxiety scores observed in severity grades 1 and 2 and the lowest mean anxiety scores observed in the worst severity group (mean 3.42). For every one grade increase in neurophysiologically assessed severity there was an estimated 0.26 point decrease in the HADS anxiety score. Using the same multivariable regression model a highly significant independent association was also found between symptom severity and hand function subscales. For every 1 point increase in symptom severity there was a marked decrease in hand function ranging from 13.4 to 17.8 points on the MHQ (see Table 4b for parameter estimates by subscales). Electrodiagnostic severity was also significantly associated with overall hand function and unilateral and bilateral activities of daily living but not with the work subscale. The magnitude of decline in hand function was less marked when using NCS grade in the model.

The mean cost of NHS service use per patient in the three months prior to baseline assessment was £447 (SD = £274), with a societal cost (NHS plus patient out of pocket and lost productivity) of £636 (SD = £694). NHS costs comprised predominantly hospital visits including NCS (£362), GP consultations (£58), prescription medications (£14) and other contacts and treatments (£13), which accounted for 70% of societal costs. Lost productivity (£101) accounted for 15%, and out of pocket costs (£56 for lost wages due to GP/hospital visits, £7 for travel to the hospital, £4 for hand splints and £18 for other contacts and treatments for the remaining 15%. Although those individuals over the age of 40 generally have higher NHS and lower societal costs compared to 18-40 year olds (reference category), age is not a significant predictor of costs under either perspective. For example, NHS costs are 15% higher on average for those individuals in the 81 plus age group, when compared to 18-40 year olds and accounting for NCS grade, but this, however, is not a significant determinant of costs ($p=0.327$). NHS and societal costs, however, significantly increase with self-

For peer review only - http://bmjopen.bmj.com/site/about/guidelines.xhtml

For BMJ Open

reported severity of CTS (p<0.01 and p<0.0001), but not NCS grade (p=0.269 and p=0.590). For every point increase in the self-reported severity, NHS and societal costs increase by 8% and 18%, respectively (see Table 5).

Discussion

Principal findings:

. We found that at the point of referral for NCS and prior to any treatment, greater symptom severity was associated with greater psychological distress, poorer hand function and lower quality of life but not with worse electrodiagnostic abnormalities. Even after adjusting for known and potential confounders such as comorbidities, age, gender and BMI, we observed that every one point increase in symptom severity score was associated with an estimated 1.11 points increase in mean anxiety score, a 1.43 point increase in depression score and a 0.10 decrease in health utility. Adjusting for the same independent variables, objectively graded severity from NCS, however, showed a significant negative association with anxiety and no significant association with depression or health utility. When modelling the outcome on self-reported hand function (MHQ) both symptom severity and NCS showed a significant association with overall hand function and activities of daily living, though the decrease in hand function was much more marked when using symptom severity (CTS-6). More than 40% of the variation in hand function was explained by the model when using CTS-6 score and less than 20% when using NCS grade in the model.

Average NHS and societal costs per patient were £447 and £636, respectively. There was no significant association between costs, age and objectively graded severity. We found, however, for every point increase in subjective severity, an 8% and 18% relative increase in NHS and societal costs, respectively.

Several other studies have explored the association of pain severity with a range of psychological variables including anxiety, depression, pain catastrophizing and coping[9 10 21]. These studies all conclude that illness behaviour is a stronger predictor of pain severity than objective measures of disease severity (NCS). In our study we did not measure pain intensity, although the CTS-6 does contain 3 items relating to pain (daytime pain, nocturnal night and waking from pain). It should also be noted that pain is not always the predominant symptom[8].

For peer review only - http://bmjopen.bmj.com/site/about/guidelines.xhtml

For BMJ Open

To the best of our knowledge this is the first study to explore the association of self-reported and objectively graded symptom severity with psychological distress and quality of life using a multivariable model whilst adjusting for the effect of other confounders. It is also the first study to measure prospectively the NHS resource use and societal costs of patients with CTS. We included a wide range of variables which could account for increased psychological distress and decreased quality of life including comorbidities weighted by how activity limiting they are, smoking status, alcohol consumption, age, gender, ethnicity, employment status and duration of CTS.

A surprising finding is that objective severity assessed by NCS showed only a weak but negative association with anxiety and there were no significant associations between NCS and HRQoL or NCS and depression. There has been much debate over the value of NCS in the diagnosis of CTS [1 22 23 24] and in the UK not all patients have NCS [7]. Several studies have examined the association of self-reported symptoms and function with objective severity from electrodiagnostic findings. Most have used simple correlations with the exception of Chan et al [25]. The latter used a multivariable model controlling for several demographic variables, as well as psychological measures including depression, somatisation and pain-related catastrophizing, in a sample of 215 patients with CTS. They conclude that subjective symptoms and objective severity are independent of each other. One possible explanation is that NCS can only measure the velocity and amplitude of large myelinated fibres that is sensory and motor axons. Yet CTS also affects the small unmyelinated fibres [26] and it is these which account for the neuropathic pain component manifesting as pain during day and night time. Only three out of the six questions in the CTS-6 address sensory symptoms (daytime numbness or tingling, night time numbness or tingling and waking from numbness and tingling) with the other three questions relating to pain at night, in the day and waking due to pain. Patients with more severe electrodiagnostic CTS (grade 5 and 6) have numbness and even muscle wasting but often do not have any 'positive' symptoms such as tingling or pain resulting in a lower overall self-reported symptom score. It has also been suggested that those with more severe compression often have less subjective symptoms, especially in older patients in whom there is a natural age-related decline in sensory function and reduced pain sensitivity [27] but greater functional disability [28]. This may also explain the significant association observed between NCS grade and hand function (MHQ) as severe numbness and thenar wasting found in grades 5 and 6 are likely to have a greater impact on hand function.

For peer review only - http://bmjopen.bmj.com/site/about/guidelines.xhtml

For BMJ Open

Another reason for the lack of association with nerve conduction results is that CTS causes small fibre loss not detected by NCS and, surprisingly, in some people with more severe nerve entrapment subjective symptoms and function improve suggesting an adaptive mechanism [26].

The slight association of anxiety with very mild or mild electrophysiological abnormalities of the median nerve CTS could represent disproportionate concern about mild symptoms from mild disease (the essence of anxiety), misdiagnosis of carpal tunnel syndrome with incidental mild electrophysiological abnormalities or a reaction to dismissive clinicians.

Our analysis, being cross-sectional, cannot address the question of whether anxiety and depression are the cause or the effect of self-reported symptom severity. There may well be a bidirectional relationship between symptom severity and psychological distress, whereby each contributes to the development and is a consequence of the other. Neither can we conclude from these data that if symptom severity improves after treatment then psychological distress or quality of life would also improve. A possible explanation for the significant independent association between self-reported symptoms, psychological status and HRQoL is that people either consistently underplay or exaggerate their symptoms, irrespective of whether these are physical, psychological or global HRQoL. This may also explain the increased costs, both from an NHS and personal perspective, driven by increased treatment-seeking behaviour in those who perceive their symptoms as worse.

Health-related QoL was markedly lower in those with very severe self-reported symptoms (score 4 to 5 on CTS-6). Their mean health utility of 0.43 is much lower than that reported in other studies of CTS patients prior to surgery ranging from 0.74 to 0.81 using the EQ-5D and 0.69 using the SF-36 or SF-6D[29-31]. In fact the mean utility index for this group is closer to that reported by EQ-5D in people with serious diseases like severe heart failure [32] moderate to severe psychotic illness [33] and digestive system cancers [34].

Strengths and limitations:

The strengths of the present study include its prospective design, multi-centre recruitment and a large sample size (>700) in whom a wide range of pre-specified sociodemographic and clinical variables were collected at baseline. Using a multivariable model mitigates the potentially confounding factors such as age, comorbidities, etc. by adjusting for these and thus testing the independence of any associations between self-reported or neurophysiological severity and psychological distress, hand function or quality of life.

For peer review only - http://bmjopen.bmj.com/site/about/guidelines.xhtml

There are also limitations, namely that the data collection was reliant on patient-self-report which is open to recall bias and data quality could not be independently verified. For example, duration of symptoms may be difficult to attribute separately to each hand in the case of bilateral symptoms or to remember accurately over a longer period. Patients were recruited through secondary care having been referred by a primary care physician. It is therefore possible that our study sample is biased towards those actively seeking treatment for their CTS. Long term follow-up over 2 years is underway and will allow us to explore predictors of treatment related outcomes and cost. We were also not able to explain why costs were higher for the lowest and highest CTS-6 grades, although it should be borne in mind that these costs relate only to the three months prior to entry in the study. The 2 year follow-up data will provide a more robust analysis. Finally our sample is biased towards an older age group and may not be representative of the typical clinical population with CTS.

Conclusions

This study shows that even when accounting for potential confounders such as, for example comorbidities or age, a strong and highly significant association remains between symptom severity and psychological status and health-related quality of life. This means that those with more severe symptoms are also more likely to present with worst mental health, poorer hand function and lower quality of life, thus placing a significant burden on the individual, health services and society. Therefore, access to timely and effective diagnostics and effective treatment before symptoms become severe or very severe is paramount. NCS remains a useful tool to assess disease severity, which in turn should be used to determine whether surgery is indicated or not, however NCS should not be used as a measure of the impact of CTS on the person. Patients presenting with only mild disease severity on NCS but who exhibit anxiety or depression may need to be offered additional psychological support.

Tables and Figures

Table 1: Demographics

Sex	*Male*	260 (35%)
	Female	493 (65%)
Age (years)	*Mean (SD)*	60.3 (12.7)
Ethnicity	*White*	731 (97%)
	Other	20 (3%)
	Missing	2
BMI	*Mean (SD)*	28.7 (5.8)

For peer review only - http://bmjopen.bmj.com/site/about/guidelines.xhtml

For BMJ Open

Smoking status	*Current*	76 (10%)
	Ex-smoker	299 (40%)
	Non-smoker	375 (50%)
	Missing	3
Alcohol intake (units)	*None*	251 (34%)
	1-4	271 (37%)
	5-10	114 (15%)
	11-20	81 (11%)
	> 21	25 (3%)
	Missing	11
Working status	*Working:*	296 (39%)
	Self-employed	88 (11.7%)
	not working:	457 (61%)
	retired	322 (43%)
	unemployed	14 (1.9%)
	long-term sick	36 (4.8%)
	carer, volunteer or student	85 (11.3%)
Income (per annum)	*< £15K*	170 (23%)
	£15K - £21.5K	103 (14%)
	£21.5 - £35K	148 (20%)
	£35K - £50K	81 (11%)
	> £50K	49 (7%)
	Missing	5

For peer review only - http://bmjopen.bmj.com/site/about/guidelines.xhtml

For peer review only

For BMJ Open

Table 2: Clinical Characteristics

Duration of CTS	<3 months	42 (6%)
	3-6 months	140 (19%)
	6-12 months	181 (24%)
	12-18 months	98 (13%)
	>18 months	292 (39%)
Side of CTS	Left	89 (12%)
	Right	148 (20%)
	Bilateral	516 (69%)
Dominant Hand Affected	Yes	669 (89%)
	No	84 (11%)
Worst Side	Left	264 (35%)
	Right	489 (65%)
Worst Side NCS Grade	1	150 (20%)
	2	79 (11%)
	3	202 (27%)
	4	137 (18%)
	5	159 (21%)
	6	24 (3%)
	missing	2
CTS-6 (1 to 5)	Mean (SD)	2.89 (0.85)
MHQ overall function (0-100)	Mean (SD)	53.4 (22.6)
MHQ unilateral activities (0-100)	Mean (SD)	66.6 (28.6)
MHQ bilateral activities (0-100)	Mean (SD)	69.4 (25.5)
MHQ work (0-100)	Mean (SD)	64.7 (25.6)
HADS Anxiety	Mean (SD)	6.18 (4.52)
	Normal (1-7)	497 (66%)
	Mild (8-11)	123 (16%)
	Moderate (11-14)	90 (12%)
	Severe (15-21)	42 (6%)
	Missing	1
HADS Depression	Mean (SD)	4.48 (3.84)
	Normal (1-7)	602 (80%)
	Mild (8-10)	88 (12%)
	Moderate (11-14)	47 (6%)
	Severe (15-21)	15 (2%)
	Missing	1
Overall Co-morbidity Score (0-36)	Mean (SD)	5.22 (4.17)
EQ5D-3L VAS	Mean (SD)	73.54 (18.2)

For peer review only

For peer review only - http://bmjopen.bmj.com/site/about/guidelines.xhtml

For BMJ Open

Table 3: Self-reported Comorbidities

Comorbidity	Has problem	Receives treatment	Limits activity
Back pain	342 (45%)	142 (19%)	226 (30%)
Osteoarthritis	253 (34%)	129 (17%)	185 (25%)
High Blood Pressure	235 (31%)	226 (30%)	22 (3%)
Depression	148 (20%)	117 (16%)	63 (8%)
Diabetes	77 (10%)	63 (8%)	11 (1%)
Rheumatoid Arthritis	70 (9%)	40 (5%)	51 (7%)
Lung disease	29 (4%)	23 (3%)	18 (2%)
Cancer	18 (2%)	13 (2%)	5 (<1%)
Kidney disease	14 (2%)	5 (<1%)	3 (<1%)
Liver disease	4 (<1%)	1 (<1%)	0
Ulcer or stomach disease	37 (5%)	32 (4%)	7 (1%)
Other Condition 1	372 (49%)	280 (37%)	150 (20%)
Other Condition 2	140 (19%)	95 (13%)	60 (8%)
Other Condition 3	48 (6%)	30 (4%)	20 (3%)

For peer review only

For peer review only - http://bmjopen.bmj.com/site/about/guidelines.xhtml

	n	*HADS ANX Mean (SD)*	*Estimate (95% CI)*[1]	*HADS DEP Mean (SD)*	*Estimate (95% CI)*[1]	*EQ-5D Mean (SD)*	*Estimate (95% CI)*[1]
CTS 6 Score							
1.00	7	3.14 (2.61)		1.57 (1.81)		0.78 (0.32)	
1.01 – 2.00	134	5.04 (4.07)		2.90 (3.12)		0.77 (0.20)	
2.01 – 3.00	308	5.50 (4.05)		3.86 (3.31)		0.72 (0.20)	
3.01 – 4.00	241	6.87 (4.55)		5.42 (3.99)		0.60 (0.28)	
4.01 – 5.00	63	9.62 (5.48)	1.11<br>(0.73, 1.49)<br>P<0.0001<br>R^2 = 17.7%	7.68 (4.44)	1.43<br>(1.13, 1.73)<br>P<0.0001<br>R^2 = 26.1%	0.43 (0.34)	-0.10<br>(-0.12, -0.08)<br>P<0.0001<br>R^2 = 37.4%
NCS Grade							
1	150	6.78 (4.79)		4.64 (4.10)		0.66 (0.28)	
2	79	7.13 (4.78)		4.78 (3.73)		0.68 (0.25)	
3	202	6.44 (4.36)		4.33 (3.77)		0.68 (0.27)	
4	137	6.24 (4.54)		4.80 (3.92)		0.68 (0.26)	
5	159	5.18 (4.24)		4.20 (3.80)		0.66 (0.24)	
6	24	3.42 (3.09)	-0.26<br>(-0.49, -0.03)<br>P=0.029<br>R^2 = 14.3%	4.08 (3.13)	-0.00<br>(-0.20, 0.20)<br>P=0.983<br>R^2 = 17.1%	0.61 (0.26)	-0.01<br>(-0.03, 0.01)<br>P=0.214<br>R^2 = 28.4%

Table 4(a) Relationship between CTS Severity and Anxiety, Depression and Quality of Life

1: Parameter estimate (95% confidence interval) from a linear model including gender, age, ethnicity, employment status, smoking status, units of alcohol per week, BMI, duration of CTS and comorbidity score.

For peer review only

For BMJ Open

Table 4(b) Relationship between CTS Severity and MHQ sub-scales

	n	Overall Function Mean (SD)	Estimate (95% CI)[1]	ADL unilateral Mean (SD)	Estimate (95% CI)[1]	ADL bilateral Mean (SD)	Estimate (95% CI)[1]	Work Mean (SD)	Estimate (95% CI)[1]
CTS 6 Score									
1.00	7	92.1 (10.7)		91.4 (16.5)		88.8 (11.4)		92.9 (12.9)	
1.01 – 2.00	134	68.5 (20.3)		82.9 (22.0)		82.9 (18.9)		78.7 (22.3)	
2.01 – 3.00	308	58.3 (19.1)		74.5 (23.1)		76.2 (20.7)		70.7 (22.8)	
3.01 – 4.00	241	44.9 (18.9)		56.8 (26.3)		60.7 (24.9)		55.0 (23.2)	
4.01 – 5.00	63	25.8 (16.2)	-14.7 (-16.4, -13.1) P<0.0001 R^2 = 41.1%	28.2 (26.4)	-17.8 (-19.9, -15.8) P<0.0001 R^2 = 40.7%	38.3 (26.2)	-14.6 (-16.4, -12.8) P<0.0001 R^2 = 40.8%	39.6 (22.2)	-13.4 (-15.4, -11.5) P<0.0001 R^2 = 33.3%
NCS Grade									
1	150	58.9 (23.9)		71.0 (27.9)		72.7 (24.4)		63.9 (27.4)	
2	79	53.5 (22.9)		71.5 (27.7)		74.1 (25.2)		67.5 (26.2)	
3	202	54.6 (22.8)		70.5 (27.7)		72.6 (24.6)		65.8 (26.0)	
4	137	53.1 (20.5)		67.0 (26.3)		68.2 (24.3)		63.4 (23.9)	
5	159	48.8 (21.2)		57.8 (30.0)		63.5 (27.3)		64.4 (24.4)	
6	24	43.3 (24.9)	-3.16 (-3.56, -1.22) P<0.0001 R^2 = 15.5%	47.9 (29.8)	-3.87 (-5.32, -2.42) P<0.0001 R^2 = 18.7%	52.5 (26.0)	-3.16 (-4.42, -1.90) P<0.0001 R^2 = 21.9%	61.9 (23.6)	-0.86 (-2.18,0.47) P=0.197 R^2 = 15.7%

[1] Parameter estimate (95% confidence interval) from a linear model including gender, age, ethnicity, employment status, smoking status, units of alcohol per week, BMI, duration of CTS and comorbidity score. ADL: activities of daily living

For peer review only

For peer review only - http://bmjopen.bmj.com/site/about/guidelines.xhtml

For BMJ Open

	n	NHS cost (SD)	exp(b)(95% CI)[1]	Societal cost (SD)	exp(b)(95% CI)[1]
**CTS-6**					
1.00	7	£512.83 (£405.61)		£726.02 (£776.80)	
1.01-2.00	134	£434.04 (£212.53)		£559.37 (£430.53)	
2.01-3.00	308	£422.57 (£220.30)		£554.73 (£492.25)	
3.01-4.00	241	£466.50 (£348.74)		£686.11 (£814.25)	
4.01-5.00	63	£511.52 (£273.65)	1.08 (1.03, 1.12)	£984.19 (£1,197.94)	1.18 (1.10, 1.27)

For peer review only

Table 5: Relationship between CTS Severity, Age and Cost

For peer review only - http://bmjopen.bmj.com/site/about/guidelines.xhtml

For BMJ Open

			**p<0.01**		**p<0.0001**
NCS Grade					
1	150	£483.36 (£456.12)		£629.69 (£608.90)	
2	79	£459.44 (£243.93)		£592.70 (£381.55)	
3	202	£438.50 (£213.31)		£657.58 (£755.06)	
4	137	£414.14 (£153.80)		£578.23 (£597.95)	
5	159	£439.38 (£203.02)		£676.24 (£892.99)	
6	24	£467.71 (£237.77)	0.98 (0.96, 1.01) p=0.269	£583.77 (£294.98)	1.01 (0.96, 1.07) P=0.590
Age & CTS-6					
Age 18 – 40	43	£433.41 (£178.60)		£758.67 (£1,099.72)	
Age 41 – 60	344	£444.53 (£226.94)	1.04 (0.88, 1.22) p=0.679	£613.03 (£533.45)	0.83 (0.63, 1.10) p=0.196
Age 61 – 80	322	£451.96 (£336.62)	1.13 (0.94, 1.36) p=0.190	£631.64 (£731.18)	0.90 (0.66, 1.22) p=0.486
Age 81 plus	44	£442.46 (£146.20)	1.16 (0.91, 1.47) p=0.224	£712.66 (£973.69)	1.09 (0.73, 1.62) p=0.690
Age & NCS Grade					
Age 18 – 40	43	£433.41 (£178.60)		£758.67 (£1,099.72)	
Age 41 – 60	343	£443.81 (£226.88)	1.02 (0.87, 1.21) p=0.774	£608.42 (£527.83)	0.81 (0.60, 1.09) p=0.163
Age 61 – 80	321	£451.29 (£336.93)	1.11 (0.93, 1.34) p=0.246	£629.87 (£730.63)	0.85 (0.61, 1.18) p=0.336
Age 81 plus	44	£442.46 (£146.20)	1.15 (0.90, 1.47) p=0.252	£712.65 (£973.69)	0.99 (0.64, 1.53) p=0.976

[1] Exponentiated parameter estimate (standard error) from a GLM model with log-link and gamma distributed errors including gender, age, ethnicity, employment status, smoking status, units of alcohol per week, BMI, bilateral CTS, co-morbidities and comorbidity score.

For peer review only

References

1. Padua L, Coraci D, Erra C, et al. Carpal tunnel syndrome: clinical features, diagnosis, and management. The Lancet Neurology;**15**(12):1273-84.
2. Bland JDP. Carpal tunnel syndrome. Curr Opin Neurol 2005;**18**(5):581-85.
3. Latinovic R, Gulliford MC, Hughes RAC. Incidence of common compressive neuropathies in primary care. J Neurol Neurosur Ps 2006;**77**(2):263-65.
4. Meys V, Thissen S, Rozeman S, et al. Prognostic factors in carpal tunnel syndrome treated with a corticosteroid injection. Muscle & nerve 2011;**44**(5):763-8.
5. Hameso A, Bland JD. Prevalence of decompression surgery in patients with carpal tunnel syndrome 8 years after initial treatment with a local corticosteroid injection. The Journal of hand surgery, European volume 2017;**42**(3):275-80.
6. Bebbington E, Furniss D. Linear regression analysis of Hospital Episode Statistics predicts a large increase in demand for elective hand surgery in England. J Plast Reconstr Aes 2015;**68**(2):243-51.
7. Ryan D, Shaw A, Graham S, et al. Variation in CCG policies for the treatment of carpal tunnel syndrome. RCS Bulletin 2017;**99**(1):28-31.
8. Duckworth AD, Jenkins PJ, Roddam P, et al. Pain and carpal tunnel syndrome. The Journal of hand surgery 2013;**38**(8):1540-6.
9. Nunez F, Vranceanu AM, Ring D. Determinants of pain in patients with carpal tunnel syndrome. Clinical orthopaedics and related research 2010;**468**(12):3328-32.
10. Fernandez-de-las-Penas C, Fernandez-Munoz JJ, Palacios-Cena M, et al. Direct and Indirect Effects of Function in Associated Variables Such as Depression and Severity on Pain Intensity in Women with Carpal Tunnel Syndrome. Pain medicine 2015;**16**(12):2405-11.
11. Jerosch-Herold C, Shepstone L, Wilson EC, et al. Clinical course, costs and predictive factors for response to treatment in carpal tunnel syndrome: the PALMS study protocol. BMC musculoskeletal disorders 2014;**15**:35.
12. Bland JDP. A neurophysiological grading scale for carpal tunnel syndrome. Muscle & nerve 2000;**23**(8):1280-83.
13. Levine D, Simmons B, Koris M, et al. A self-administered questionnaire for the assessment of severity of symptoms and functional status in carpal tunnel syndrome. The Journal of Bone and Joint Surgery 1993;**75A**(11):1585-92.
14. Atroshi I, Lyren PE, Gummesson C. The 6-item CTS symptoms scale: a brief outcomes measure for carpal tunnel syndrome. Qual Life Res 2009;**18**(3):347-58.
15. Chung KC, Pillsbury MS, Walters MR, et al. Reliability and validity testing of the Michigan Hand Outcomes Questionnaire. J Hand Surg-Am 1998;**23A**(4):575-87.
16. Kotsis SV, Chung KC. Responsiveness of the Michigan Hand Outcomes Questionnaire and the Disabilities of the Arm, Shoulder and Hand Questionnaire in carpal tunnel surgery. J Hand Surg-Am 2005;**30A**(1):81-86.
17. Snaith RP, Zigmond AS. The Hospital Anxiety and Depression Scale. Brit Med J 1986;**292**(6516):344-44.
18. Group E. EuroQol-a new facility for the measurement of health-related quality of life Health Policy 1990;**16**(3):199-208.
19. Sangha O, Stucki G, Liang MH, et al. The self-administered comorbidity questionnaire: A new method to assess comorbidity for clinical and health services research. Arthrit Rheum-Arthr 2003;**49**(2):156-63.
20. Dolan P. Modeling valuations for EuroQol health states. Med Care 1997;**35**(11):1095-108.
21. Hobby JL, Venkatesh R, Motkur P. The effect of psychological disturbance on symptoms, self-reported disability and surgical outcome in carpal tunnel syndrome. J Bone Joint Surg Br 2005;**87B**(2):196-200.

For peer review only - http://bmjopen.bmj.com/site/about/guidelines.xhtml

For BMJ Open

22. Sears ED, Swiatek PR, Hou H, et al. Utilization of Preoperative Electrodiagnostic Studies for Carpal Tunnel Syndrome: An Analysis of National Practice Patterns. J Hand Surg-Am 2016;**41**(6):665-72.
23. Thomson JG. Diagnosis and treatment of carpal tunnel syndrome -correspondence. The Lancet Neurology;**16**(4):263.
24. Padua L, Coraci D, Erra C, et al. Diagnosis and treatment of carpal tunnel syndrome - Authors' response. The Lancet Neurology;**16**(4):263-64.
25. Chan L, Turner JA, Comstock BA, et al. The relationship between electrodiagnostic findings and patient symptoms and function in carpal tunnel syndrome. Archives of physical medicine and rehabilitation 2007;**88**(1):19-24.
26. Schmid AB, Bland JDP, Bhat MA, et al. The relationship of nerve fibre pathology to sensory function in entrapment neuropathy. Brain 2014;**137**:3186-99.
27. Povlsen B, Aggelakis K, Koutroumanidis M. Effect of age on subjective complaints and objective severity of carpal tunnel syndrome: prospective study. JRSM short reports 2010;**1**(7):62.
28. Hobby JL, Venkatesh R, Motkur P. The effect of age and gender upon symptoms and surgical outcomes in carpal tunnel syndrome. J Hand Surg-Brit Eur 2005;**30B**(6):599-604.
29. Nazari G, MacDermid JC, Bain J, et al. Estimation of health-related-quality of life depends on which utility measure is selected for patients with carpal tunnel syndrome. Journal of hand therapy : official journal of the American Society of Hand Therapists 2016.
30. Marti C, Hensler S, Herren DB, et al. Measurement properties of the EuroQoL EQ-5D-5L to assess quality of life in patients undergoing carpal tunnel release. The Journal of hand surgery, European volume 2016.
31. Atroshi I, Gummesson C, McCabe SJ, et al. The SF-6D health utility index in carpal tunnel syndrome. The Journal of hand surgery, European volume 2007;**32**(2):198-202.
32. Dyer MT, Goldsmith KA, Sharples LS, et al. A review of health utilities using the EQ-5D in studies of cardiovascular disease. Health and quality of life outcomes 2010;**8**(1):13.
33. Hakkaart L, Lamers LM, Bouwmans CAM, et al. Comparison of EQ-5D and SF-6D in mental health patients. J Ment Health Policy 2007;**10**:S17-S17.
34. Pickard AS, Wilke CT, Lin HW, et al. Health utilities using the EQ-5D in studies of cancer. Pharmacoeconomics 2007;**25**(5):365-84.

Figure Legends

Figure 1 : STROBE flowchart

For peer review only

Figure 1 : STROBE flowchart [31]

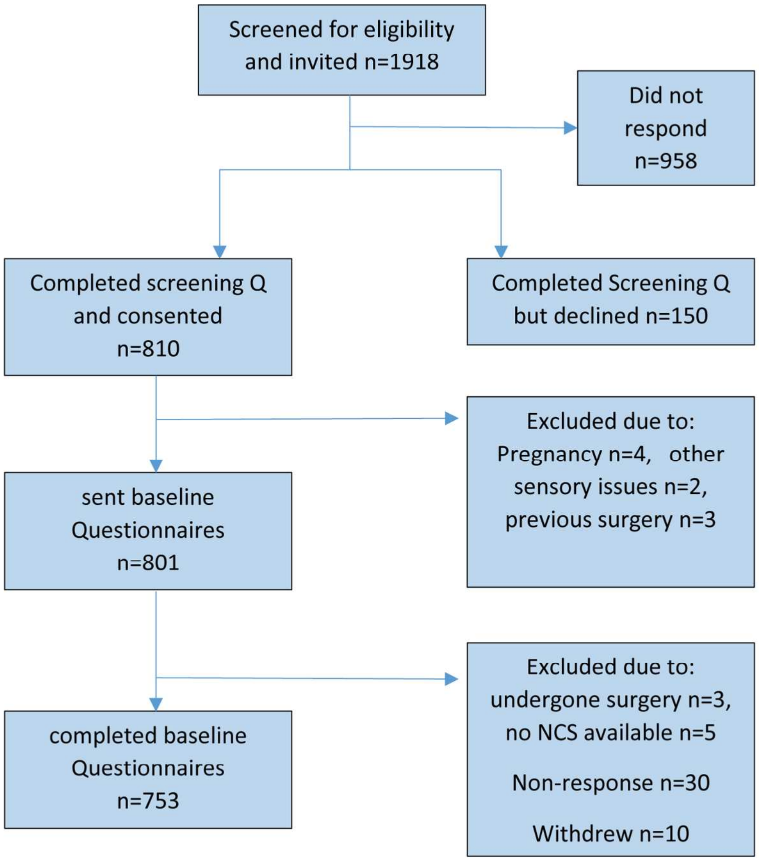

Figure1: STROBE flowchart

156x142mm (300 x 300 DPI)

Supplementary File I:

Criteria for grading of nerve conduction studies:

Grade 0	Normal	No neurophysiological abnormality
Grade 1	Very mild	Median SAP digit II or III to wrist: < 50m/s and ≥40 m/s, Ulnar SAP>50m/s<br>Median DML APB to wrist: >4.0ms and Ulnar DML <=3.8ms<br>or<br>2 sensitive tests positive (ring finger 'double peak' SAP or palm/wrist – median/ulnar comparison or Lumbrical to interosseous latency increase)
Grade 2	Mild	Median SAP digit II or III to wrist SAP: < 40 m/sec<br>Median DML APB to wrist: < 4.5msec
Grade 3	Moderate	Median SAP digit II or III to wrist: present<br>Median DML APB to wrist: > 4.5msec and < 6.5 ms
Grade 4	Severe	Median SAP digit II or III to wrist: absent<br>Median DML APB to wrist: > 4.5 and < 6.5 ms
Grade 5	Very severe	Median DML APB to wrist : > 6.5 msec
Grade 6	Extremely severe	Median DML APB to wrist: unrecordable (< 0.2 mV peak to peak)

SAP: sensory action potential, DML: distal motor latency; APB: Abductor Pollicis Brevis; ms: milliseconds

For peer review only

For peer review only

STROBE Statement—checklist of items that should be included in reports of observational studies

	Item No	Recommendation	Page
**Title and abstract**	1	(*a*) Indicate the study's design with a commonly used term in the title or the abstract	**3**
		(*b*) Provide in the abstract an informative and balanced summary of what was done and what was found	**3**
**Introduction**			
Background/rationale	2	Explain the scientific background and rationale for the investigation being reported	**4**
Objectives	3	State specific objectives, including any prespecified hypotheses	**4**
**Methods**			
Study design	4	Present key elements of study design early in the paper	**3/4**
Setting	5	Describe the setting, locations, and relevant dates, including periods of recruitment, exposure, follow-up, and data collection	**4/5**
Participants	6	(*a*) *Cohort study*—Give the eligibility criteria, and the sources and methods of selection of participants. Describe methods of follow-up<br>*Case-control study*—Give the eligibility criteria, and the sources and methods of case ascertainment and control selection. Give the rationale for the choice of cases and controls<br>*Cross-sectional study*—Give the eligibility criteria, and the sources and methods of selection of participants	**5**
		(*b*) *Cohort study*—For matched studies, give matching criteria and number of exposed and unexposed<br>*Case-control study*—For matched studies, give matching criteria and the number of controls per case	
Variables	7	Clearly define all outcomes, exposures, predictors, potential confounders, and effect modifiers. Give diagnostic criteria, if applicable	**5/6**
Data sources/ measurement	8*	For each variable of interest, give sources of data and details of methods of assessment (measurement). Describe comparability of assessment methods if there is more than one group	**6**
Bias	9	Describe any efforts to address potential sources of bias	**6**
Study size	10	Explain how the study size was arrived at	**n/a**
Quantitative variables	11	Explain how quantitative variables were handled in the analyses. If applicable, describe which groupings were chosen and why	**6**
Statistical methods	12	(*a*) Describe all statistical methods, including those used to control for confounding	**6**
		(*b*) Describe any methods used to examine subgroups and interactions	**6**
		(*c*) Explain how missing data were addressed	**6**
		(*d*) *Cohort study*—If applicable, explain how loss to follow-up was addressed<br>*Case-control study*—If applicable, explain how matching of cases and controls was addressed<br>*Cross-sectional study*—If applicable, describe analytical methods taking account of sampling strategy	**5/6**
		(*e*) Describe any sensitivity analyses	

For peer review only - http://bmjopen.bmj.com/site/about/guidelines.xhtml

Results			page
Participants	13*	(a) Report numbers of individuals at each stage of study—eg numbers potentially eligible, examined for eligibility, confirmed eligible, included in the study, completing follow-up, and analysed	**6**
		(b) Give reasons for non-participation at each stage	
		(c) Consider use of a flow diagram	**Fig 1**
Descriptive data	14*	(a) Give characteristics of study participants (eg demographic, clinical, social) and information on exposures and potential confounders	**12**
		(b) Indicate number of participants with missing data for each variable of interest	**12**
		(c) *Cohort study*—Summarise follow-up time (eg, average and total amount)	**n/a**
Outcome data	15*	*Cohort study*—Report numbers of outcome events or summary measures over time	
		Case-control study—Report numbers in each exposure category, or summary measures of exposure	
		Cross-sectional study—Report numbers of outcome events or summary measures	**12/13**
Main results	16	(*a*) Give unadjusted estimates and, if applicable, confounder-adjusted estimates and their precision (eg, 95% confidence interval). Make clear which confounders were adjusted for and why they were included	**7, 15, 16**
		(*b*) Report category boundaries when continuous variables were categorized	**n/a**
		(*c*) If relevant, consider translating estimates of relative risk into absolute risk for a meaningful time period	**n/a**
Other analyses	17	Report other analyses done—eg analyses of subgroups and interactions, and sensitivity analyses	
**Discussion**			
Key results	18	Summarise key results with reference to study objectives	**8**
Limitations	19	Discuss limitations of the study, taking into account sources of potential bias or imprecision. Discuss both direction and magnitude of any potential bias	**10**
Interpretation	20	Give a cautious overall interpretation of results considering objectives, limitations, multiplicity of analyses, results from similar studies, and other relevant evidence	**10**
Generalisability	21	Discuss the generalisability (external validity) of the study results	**10**
**Other information**			
Funding	22	Give the source of funding and the role of the funders for the present study and, if applicable, for the original study on which the present article is based	**1**

*Give information separately for cases and controls in case-control studies and, if applicable, for exposed and unexposed groups in cohort and cross-sectional studies.

Note: An Explanation and Elaboration article discusses each checklist item and gives methodological background and published examples of transparent reporting. The STROBE checklist is best used in conjunction with this article (freely available on the Web sites of PLoS Medicine at http://www.plosmedicine.org/, Annals of Internal Medicine at http://www.annals.org/, and Epidemiology at http://www.epidem.com/). Information on the STROBE Initiative is available at www.strobe-statement.org.

For peer review only
